# Two-Component Response Regulator OmpR Regulates Mucoviscosity through Energy Metabolism in Klebsiella pneumoniae

**DOI:** 10.1128/spectrum.00544-23

**Published:** 2023-04-25

**Authors:** Lijun Wang, Xueting Huang, Qian Jin, Jie Tang, Hua Zhang, Jing-Ren Zhang, Hui Wu

**Affiliations:** a Center for Infectious Disease Research, Department of Basic Medical Science, School of Medicine, Tsinghua University, Beijing, China; b Department of Laboratory Medicine, Beijing Tsinghua Changgung Hospital, School of Clinical Medicine, Tsinghua University, Beijing, China; c Tsinghua-Peking Center for Life Sciences, Tsinghua University, Beijing, China; d Department of Integrative Biomedical and Diagnostic Sciences, Oregon Health and Science University School of Dentistry, Portland, Oregon, USA; The Ohio State University Division of Biosciences

**Keywords:** *Klebsiella pneumoniae*, hypermucoviscosity, OmpR, F-type ATP synthase, glycine cleavage system

## Abstract

Hypermucoviscosity is a hallmark of hypervirulent Klebsiella pneumoniae (hvKP). However, the molecular basis of its regulation is largely unknown. We hypothesize that hypermucoviscosity is modulated via two-component signal transduction systems (TCSs). In-frame deletion mutants of all 33 response regulators of hvKP ATCC43816 were generated using CRISPR/CAS and evaluated for their impacts on hypermucoviscosity. The response regulator OmpR is required for hypermucoviscosity *in vitro* and virulence *in vivo* in a mouse pneumonia model. The Δ*ompR* mutant lost its mucoidy but retained its capsule level and comparable *rmpADC* expression, so transcriptomic analysis by RNA-Seq was performed to identify differentially expressed genes (DEGs) in Δ*ompR* mutant. The top 20 Gene Ontology terms of 273 DEGs belong to purine ribonucleotide triphosphate biosynthetic and metabolic process, transmembrane transport, and amino acid metabolism. Among the overexpressed genes in the Δ*ompR* mutant, the *atp* operon encoding F-type ATP synthase and the *gcvTHP* encoding glycine cleavage system were characterized further as overexpression of either operon reduced the mucoviscosity and increased the production of ATP. Furthermore, OmpR directly bound the promoter region of the *atp* operon, not the *gcvTHP*, suggesting that OmpR regulates the expression of the *atp* operon directly and *gcvTHP* indirectly. Hence, the loss of OmpR led to the overexpression of F-type ATP synthase and glycine cleavage system, which altered the energetic status of Δ*ompR* cells and contributed to the subsequent reduction in the mucoviscosity. Our study has uncovered a previously unknown regulation of bacterial metabolism by OmpR and its influence on hypermucoviscosity.

**IMPORTANCE** Hypermucoviscosity is a critical virulent factor for Klebsiella pneumoniae infections, and its regulation remains poorly understood at the molecular level. This study aims to address this knowledge gap by investigating the role of response regulators in mediating hypermucoviscosity in K. pneumoniae. We screened 33 response regulators and found that OmpR is essential for hypermucoviscosity and virulence of K. pneumoniae in a mouse pneumonia model. Transcriptomic analysis uncovered that genes involved in energy production and metabolism are highly upregulated in the Δ*ompR* mutant, suggesting a potential link between bacterial energy status and hypermucoviscosity. Overexpression of those genes increased production of ATP and reduced mucoviscosity, recapitulating the Δ*ompR* mutant phenotype. Our findings provide new insights into the regulation of K. pneumoniae hypermucoviscosity by a two-component signal transduction system, highlighting the previously unknown role of OmpR in regulating bacterial energy status and its influence on hypermucoviscosity.

## INTRODUCTION

Hypervirulent Klebsiella pneumoniae (hvKP) has become a dominant pathotype of K. pneumoniae worldwide since it was first reported in the 1980s in Taiwan (1, 2). Epidemiologic studies have revealed that hvKP strains are capable of metastatic to distant sites, including lung, liver, kidney, spleen, fascia, eye, and center nervous system (3). The identification of hvKP is often based on its clinical presentations in the infected host and its ability to infect healthy individuals systematically. However, many cases of hvKP infection are highly variable and difficult to identify and treat promptly, underscoring the urgent need for reliable biomarkers.

Several genotypic and phenotypic virulence biomarkers of hvKP have been identified, including K1/K2 serotype capsule polysaccharides, putative metabolite transporter gene *peg-344*, aerobactin synthesis gene *iucA*, salmochelin synthesis gene *iroB*, mucoid phenotype regulators *rmpADC*, and hypermucoviscosity (4–6). However, each of these biomarkers has limitations when it comes to highly varied clinical hvKP isolates that are distinct from classic KP (cKP). Hypermucoviscosity, in particular, has been regarded as a unique visual feature of hvKP, with high accuracy in predicting its presence (4, 7). Patients infected with hypermucoviscous strains tend to have a distinct invasive syndrome compared to those infected with nonhypermucoviscosity variants (8, 9). Thus, hypermucoviscosity is a promising and reliable biomarker of hvKP.

Hypermucoviscosity is a unique phenotype that distinguishes hvKP from other encapsulated strains, such as cKP, Escherichia coli, Acinetobacter baumannii, and Pseudomonas aeruginosa. Unlike the coalesce and watery mucoid colonies of these other strains, hypermucoviscous colonies form a viscous string of more than 5 mm in length when stretched with a loop from a blood agar plate (7). Abundant capsular polysaccharides and the regulator of mucoid phenotype (RmpA) are well-known determinants of hypermucoviscosity (10, 11). However, recent studies have identified additional factors that are required for hypermucoviscosity, including RmpD and central metabolism (12, 13). Thus, a further in-depth research is needed to uncover the underlying mechanisms involved in the formation of hypermucoviscosity.

Under favorable environmental conditions, non mucoid parent strains of various bacteria, including Klebsiella spp., Salmonella spp., E. coli, Vibrio parahaemolyticus, and P. aeruginosa, can evolve into mucoid variants (14, 15). Two putative determinants in hypermucoviscosity, capsule biosynthesis and RmpA regulation in hvKP, are related to RcsBCD, a two-component signal transduction system (TCS) (16, 17). Based on this, we hypothesize that hvKP develops the hypermucoviscous phenotype in response to environmental cues via TCS, which is a common regulation mechanism used by many bacteria (18). TCS can regulate the expression of capsules or lipopolysaccharide (LPS) modification to modulate bacterial virulence. For instance, AlgZ/AlgR in P. aeruginosa is required for alginate production under osmolarity and nitrate signals (19, 20). BfmR/BfmS in A. baumannii enhances capsule genes expression in response to antibiotics to confer virulence (21); EnvZ/OmpR regulates Vi polysaccharide synthesis in S. Typhi (22), and PmrA/PmrB regulates LPS modification to acquire polymyxin B resistance in K. pneumoniae (23). Therefore, the systematical study of TCSs can help uncover the molecular regulation mechanisms regulating hypermucoviscosity in hvKP.

In this study, we investigated the effects of 33 response regulators on hypermucoviscosity in hvKP ATCC43816 and discovered that OmpR is essential for both hypermucoviscosity and virulence. The OmpR governs hypermucoid characteristic independently of the capsule and RmpD. Transcriptomic analysis revealed that the absence of OmpR resulted in the overexpression of F-type ATP synthase and the glycine cleavage system, which in turn affects the cellular energy status and contributes to the subsequent inhibition of mucoviscosity. Our findings present a previously unknown regulatory role of OmpR in bacterial energy and its impact on hypermucoviscosity.

## RESULTS

### Response regulator OmpR is required for hypermucoviscosity.

To systematically investigate the impacts of the TCSs on mucoviscosity, we first predicted TCS homologs using the Prokaryotic 2-Component Systems (P2CS) (http://www.p2cs.org/) (24), an integrated and comprehensive database of TCS proteins. We identified 33 genes encoding TCS response regulators in the K. pneumoniae ATCC43816 genome. Subsequently, we constructed markerless in-frame deletion mutants for all 33 response regulator genes by the CRISPR-Cas9 system, as described (25). The morphology characteristics of all the isogenic mutants are listed in Table 1. Moreover, we determined the hypermucoviscosity of each isogenic strain using the string test.

**Table 1:** The characteristics of in-frame deletion mutants of 33 response regulators of hvKP ATCC43816

Strain	Genotype	Name of deleted gene	String test	Growth curve	Morphology
TH14397	Δ*VK055*_0032	*uvrY*	Positive	Normal	Typical
TH14423	Δ*VK055*_0233	*narL*	Positive	Normal	Typical
TH14429	Δ*VK055*_0249	*rssB*	Positive	Normal	Typical
TH14411	Δ*VK055*_0682	0682	Positive	Normal	Typical
TH14412	Δ*VK055*_0953	*dcuR*	Positive	Normal	Typical
TH14413	Δ*VK055*_0985	*rstA*	Positive	Normal	Typical
TH14415	Δ*VK055*_1326	*phoP*	Positive	Normal	Typical
TH14416	Δ*VK055*_1598	1598	Positive	Normal	Typical
TH14428	Δ*VK055*_1724	*pmrA*	Positive	Normal	Typical
TH14417	Δ*VK055*_1819	*kdpE*	Positive	Normal	Typical
TH14424	Δ*VK055*_2216	*phoB*	Positive	Normal	Typical
TH14398	Δ*VK055*_2521	2521	Positive	Normal	Typical
TH16060	Δ*VK055*_2547	2547	Positive	Normal	Typical
TH14426	Δ*VK055*_2575	*arcA*	Positive	Defect	Elongation
TH14418	Δ*VK055*_2578	*creB*	Positive	Normal	Typical
TH14419	Δ*VK055*_2704	*cusR*	Positive	Normal	Typical
TH14402	Δ*VK055*_2984	2984	Positive	Normal	Typical
TH14401	Δ*VK055*_3087	*zraR*	Positive	Normal	Typical
TH14420	Δ*VK055*_3257	*cpxR*	Positive	Normal	Typical
TH14406	Δ*VK055*_3297	*ntrC*	Positive	Normal	Typical
TH14421	Δ*VK055*_3393	*uhpA*	Positive	Normal	Typical
TH14427	Δ*VK055*_3696	*ompR*	Negative	Normal	Typical
TH14410	Δ*VK055*_3990	*evgA*	Positive	Normal	Typical
TH14400	Δ*VK055*_4034	*qseB*	Positive	Normal	Typical
TH16062	Δ*VK055*_4175	4175	Positive	Normal	Typical
TH14425	Δ*VK055*_4178	4178	Positive	Normal	Typical
TH16064	Δ*VK055*_4202	*evgA*	Positive	Normal	Typical
TH14409	Δ*VK055*_4622	*yfhA*	Positive	Normal	Typical
TH14422	Δ*VK055*_4775	*mrkE*	Positive	Normal	Typical
TH14399	Δ*VK055*_4883	*rcsB*	Negative	Normal	Typical
TH14408	Δ*VK055*_4961	*btsR*	Positive	Normal	Typical
TH14404	Δ*VK055*_4995	*baeR*	Positive	Normal	Typical
TH14403	Δ*VK055*_5004	5004	Positive	Normal	Typical

Among the 33 response regulator deletion mutants, only Δ*ompR* and Δ*rcsB* mutants lost the hypermucoviscous phenotype, as indicated by a negative string test (Table 1). These findings suggest that response regulators RcsB and OmpR are required for hypermucoviscosity. RcsB (regulator of capsule synthesis) has been documented as a mucoviscosity determinant via the activation of capsule biosynthesis (10, 26). However, the mechanism by which OmpR mediates mucoviscosity remains to be elucidated.

**FIG 1:**
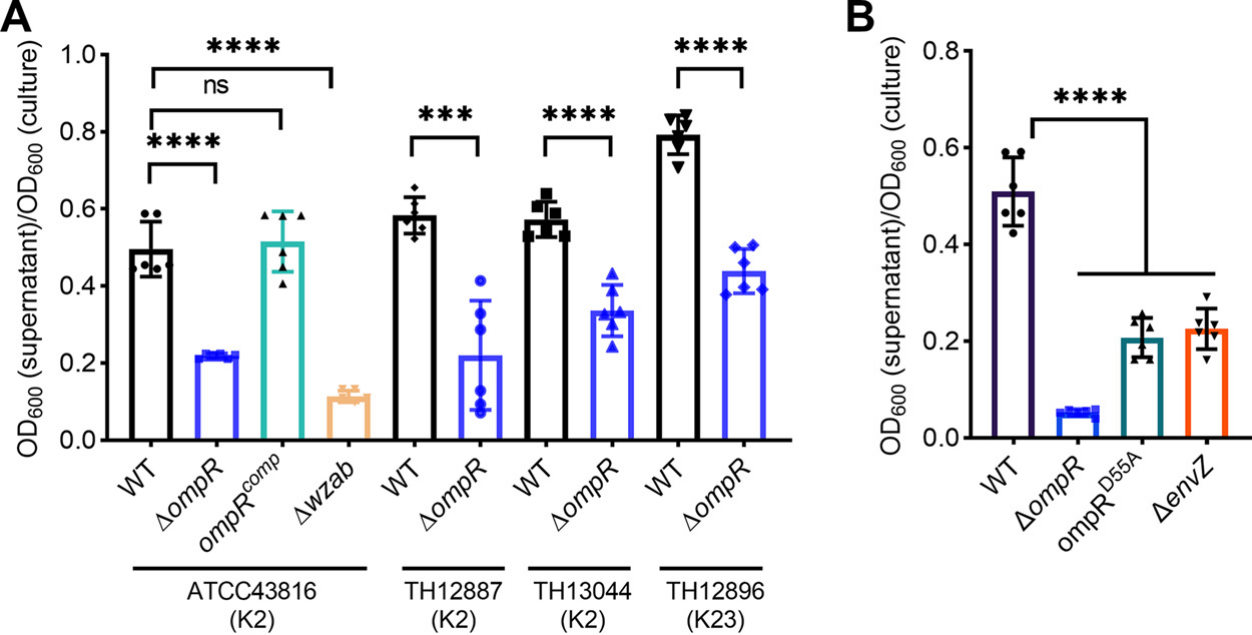
OmpR is required for hypermucoviscosity of K. pneumoniae. (A) OmpR deficiency reduced hypermucoviscosity in three K2 serotype strains (ATCC43816, TH12887, and TH13044) and one serotype K23 strain (TH12896). The *omp*R^comp^ is the complementation of the Δ*ompR* mutant, and Δ*wzab* capsule-null mutant is a negative control. (B) Effects of phosphorylation of OmpR on K. pneumoniae mucoviscosity. The *ompR*^D55A^ encodes the unphosphorylated form of OmpR, and Δ*envZ* is a histidine kinase EnvZ deletion mutant. Data are presented as mean ± SD from six biological replicates. Two-tailed *t* test and one-way ANOVA with Dunnett’s multiple-comparison test were performed to determine the statistical significance of two group comparisons. ***, *P* < 0.001; ****, *P* < 0.0001; ns, no significance.

To assess the role of OmpR in hypermucoviscosity formation, we investigated its effects in three additional hypermucoviscous clinical strains, including two serotype K2 strains (TH12887 and TH13044) and a serotype K23 strain (TH12896), by deleting the *ompR* gene using the CRISPR-Cas9 system. Similarly, the mucoviscosity of all Δ*ompR* mutants (TH16268, TH16270, and TH16070) from these strains was significantly reduced compared to the corresponding parent strains (Fig. 1A).

EnvZ/OmpR constitutes a two-component signal transduction system that controls a wide range of gene expression in response to osmotic signals or acid stress in many species of Enterobacteriaceae (27, 28). OmpR can not only act canonically (requiring phosphorylation) to regulate the porin genes but also noncanonically (without phosphorylation) to activate the acid stress response (29). Given that a single amino acid substitution of Aspirate to Alanine (D55A) mimics the unphosphorylated form of OmpR (30), we constructed a site-directed mutant *ompR*^D55A^ (TH14495) and a markerless deletion mutant of *envZ* (TH14889) to evaluate whether OmpR phosphorylation and EnvZ are required for hypermucoviscosity.

The mucoviscosity of Δ*envZ* and unphosphorylated form *ompR*^D55A^ decreased compared to that of the wild-type strain (Fig. 1B). Notably, Δ*ompR* completely lost mucoviscosity (sedimentation value < 0.2), while *ompR*^D55A^ and EnvZ deficiency reduced mucoviscosity to a lesser extent (sedimentation value 0.2 to 0.4). These data suggest that OmpR phosphorylation and EnvZ/OmpR phosphoryl relay partially account for hypermucoviscosity.

### OmpR contributes to virulence in a mouse model of pneumonia.

**FIG 2:**
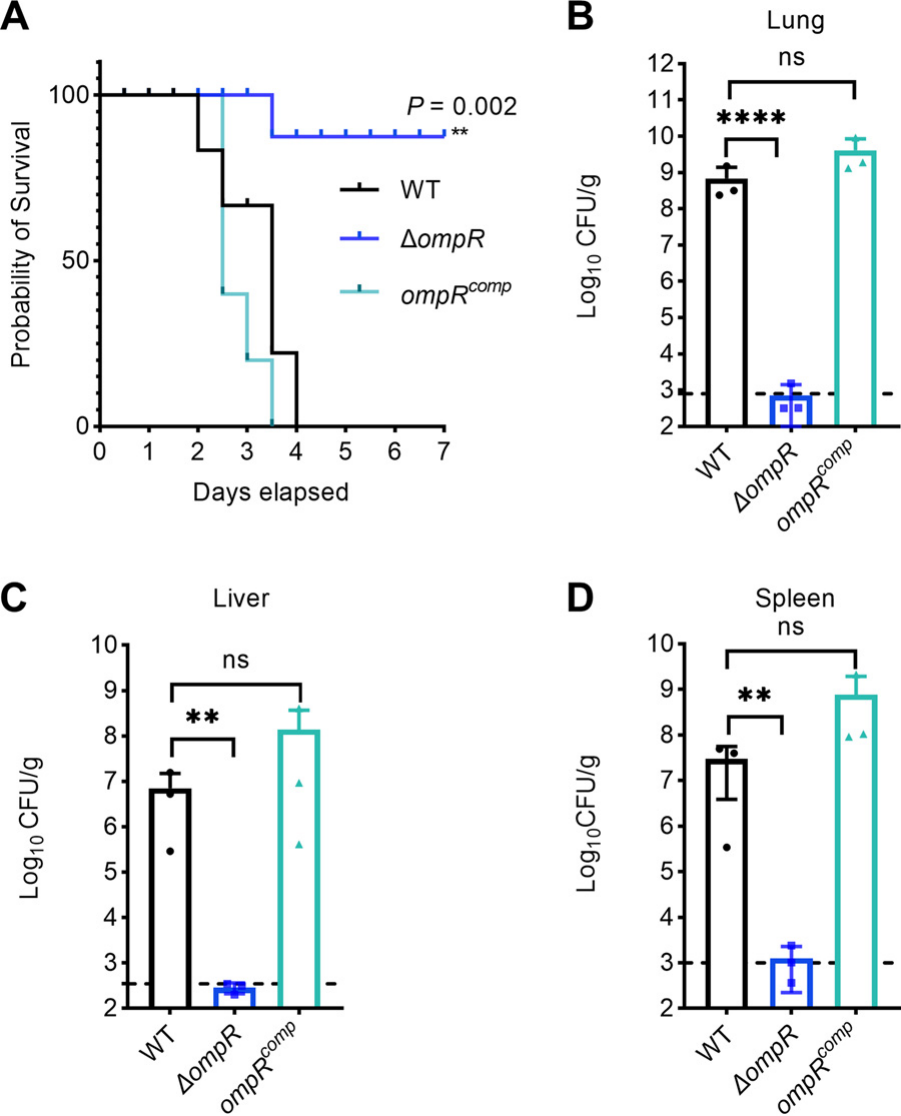
OmpR contributes to virulence *in vivo* in a mouse model of pneumonia. (A) Survival of mice infected with K. pneumoniae variants observed for 7 days postinfection (*n* = 5). Gehan-Breslow-Wilcoxon test was performed. After 72 h postinoculation, the lungs (B), livers (C), and spleens (D) were harvested for bacterial enumeration. Each circle represents one mouse, and dotted lines represent the detection limit. One-way ANOVA with Dunnett’s multiple-comparison test was performed to determine the statistical significance of two group comparisons. **, *P* < 0.01; ****, *P* < 0.0001; ns, no significance.

To determine whether mucoviscosity alterations by OmpR influences virulence, we assessed the virulence of isogenic strains in a pneumonia model with an intranasal inoculation of 2,000 CFU log-phase grown bacteria. We first evaluated virulence by examining the mortality rate postinfection. The mortality of the Δ*ompR* mutant significantly decreased compared with that of the wild-type strain (*P* < 0.01) (Fig. 2A).

As the clinical definition of hvKP involves the ability to disseminate to multiple organs, we further assessed the bacteria burden in various organs, including the lung, liver, and spleen, at 72 h postinfection. The wild-type bacteria colonized as high as 1 × 10^9^ CFU/g in the primary lung lesion and up to 1 × 10^7^ CFU/g in metastatic sites (liver and spleen). In contrast, the Δ*ompR* mutant was barely or not at all detectable in these organs (Fig. 2B to D). These data suggest that the Δ*ompR* mutant, with reduced mucoviscosity, attenuates the virulence of K. pneumoniae *in vivo*.

### Effects of OmpR on capsule production and *rmpADC* expression.

Capsule biosynthesis and RmpADC expression contribute to mucoviscosity (10, 13). Accordingly, we assessed whether OmpR regulates either capsule production or *rmp*ADC expression to determine the underlying regulatory mechanisms.

**FIG 3:**
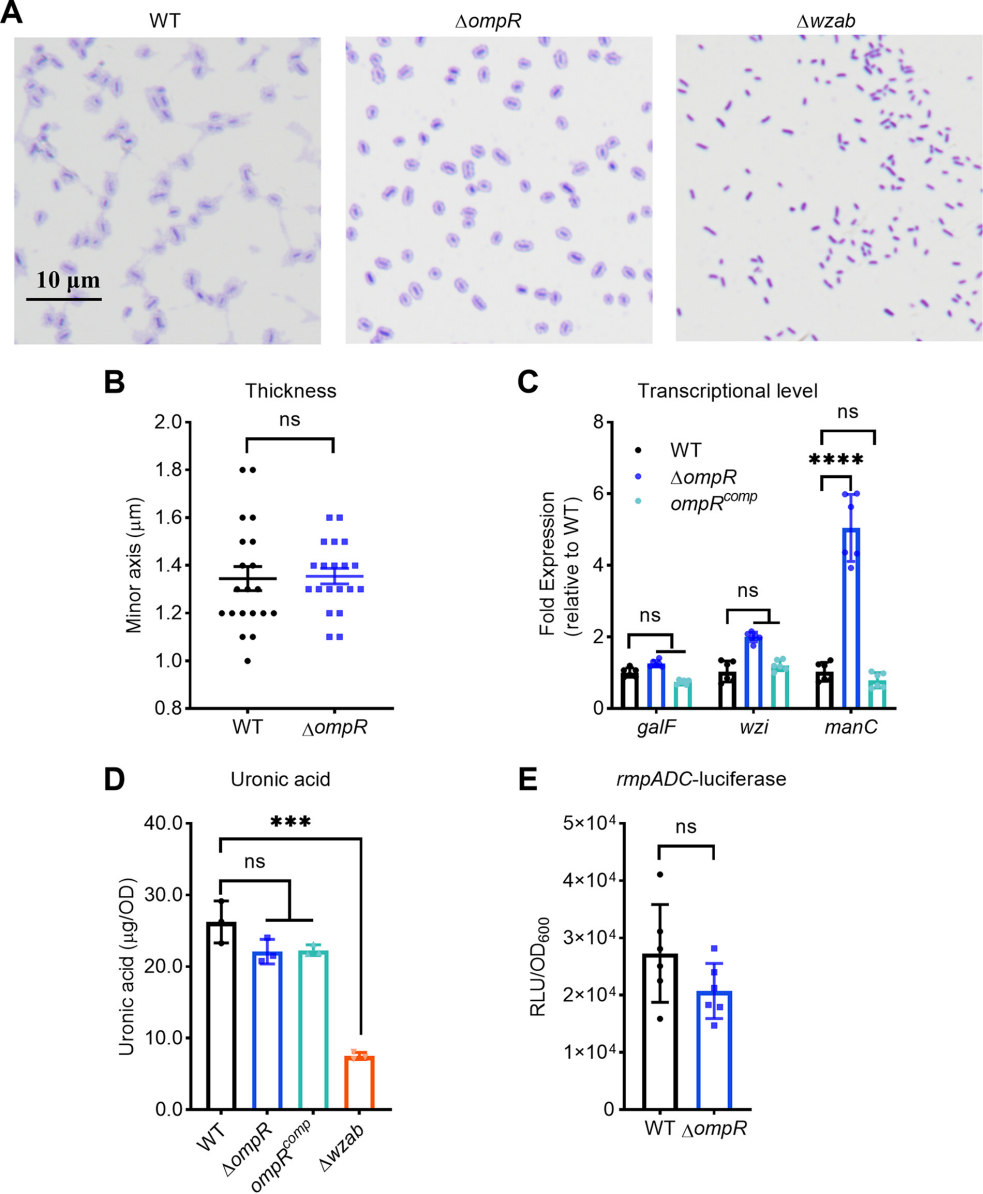
Effects of OmpR on capsule and *rmpADC* expression. Capsules were visualized by Anthony direct-dry staining (A), and the capsule thickness was quantified by measuring the minor axis of the pale blue zone surrounding 20 random cells using Image J (B). Expression of representative capsule genes was determined by quantitative RT-PCR analysis (C) (expression of each gene in the isogenic strains was normalized to that of wild-type strain), and capsule production was assessed by measuring uronic acid content (D). The *rmpADC* expression levels were monitored using a luciferase transcriptional reporter (E). Relative luminescence units (RLU) were normalized to the OD_600nm_ of culture. The *omp*R^comp^ is the complementation of the Δ*ompR* mutant, and Δ*wzab* capsule-null mutant is a negative control. Data are presented as mean ± SD from six biological replicates. Two-tailed *t*-tests were performed to determine the statistical significance of two group comparisons. ***, *P* < 0.001; ****, *P* < 0.0001; ns, no significance.

We evaluated capsule production in three aspects in K. pneumoniae ATCC43816: capsular thickness, gene expression, and uronic acid content (10). The Δ*ompR* mutant exhibited the same capsule appearance and thickness as the wild-type strain using the Anthony direct-dry staining method (Fig. 3A and B). We further determined the expression of three representative capsule synthesis genes (*galF*, *wzi*, and *manC*) (31). Expression of *galF* and *wzi* remained unchanged, while the expression of *manC* increased about 5-fold in the Δ*ompR* mutant compared to the wild-type strain (Fig. 3C). Additionally, we detected the same level of uronic acid content in both wild-type and the Δ*ompR* mutant (Fig. 3D). Thus, alternation in capsule production is not responsible for OmpR-mediated hypermucoviscosity.

Furthermore, we investigated *rmpADC* expression using luciferase reporter assays. Both wild-type and Δ*ompR* strains displayed similar reporter activities, suggesting that OmpR had no significant impact on *rmpADC* expression (Fig. 3E). Collectively, these findings suggest that the OmpR-mediated mucoid phenotype is not regulated through changes in capsule production or *rmpADC* expression, two known determinants, indicating of an unknown mechanism involved.

### Effects of OmpR on metabolic pathways of K. pneumoniae.

The response regulator OmpR has been extensively studied as a global regulatory factor, directly controlling the transcription of numerous genes in E. coli and Salmonella *typhimurium* (32). Although OmpR is identical in both species, only 15 target genes of OmpR overlap between them (33). The OmpR regulon in K. pneumoniae has not been fully explored. To investigate the OmpR regulon involved in mucoviscosity, we determined the transcriptional profiles of wild-type and Δ*ompR* cells using RNA sequencing.

**FIG 4:**
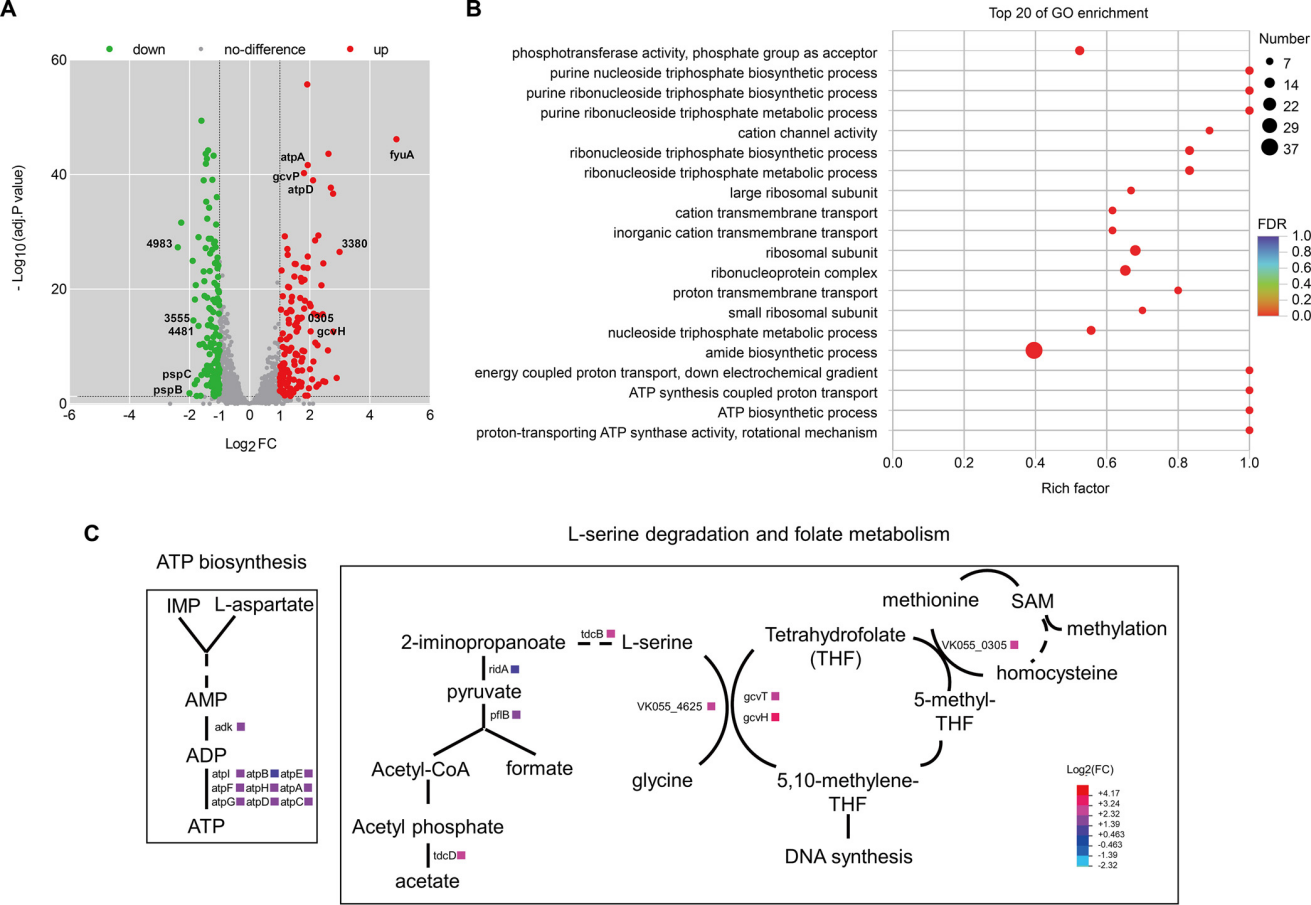
Transcriptomic analysis of OmpR-mediated gene expression. Differential gene expression between wild-type K. pneumoniae and its Δ*ompR* mutant was determined by RNA-sequencing. (A) Volcano plot analysis of differentially expressed genes. A total of 273 differentially expressed genes (DEGs), including 136 upregulated and 137 downregulated genes in Δ*ompR*, are highlighted. Representative genes are depicted in Table 2 (name or ID). The red and green dots denote significantly upregulated and downregulated genes, respectively, and the gray dots represent not differentially expressed genes. (B) The top 20 GO terms were identified by Gene Ontology analysis. (C) Metabolic pathways associated with DEGs were determined by an integrated Pathway Tools software V26.0.

We identified a total of 273 differentially expressed genes (DEGs) between wild-type and Δ*ompR* strains, comprising 136 upregulated and 137 repressed genes in Δ*ompR* (Fig. 4A). We performed Gene Ontology (GO) enrichment analysis and identified the top 20 GO terms for DEGs, which include purine ribonucleotide triphosphate biosynthesis, metabolic processes, transmembrane transport, and amino acid metabolism. (Fig. 4B). These results suggest that cellular metabolism plays a pivotal role in mucoviscosity. Consequently, we integrated all DEGs into metabolic pathways to identify underlying molecular mechanisms using the Pathway Tools software v26.0 (34, 35). The DEGs are primarily associated with metabolic pathways such as ATP *de novo* biosynthesis, l-serine, glycine, and methionine metabolism (Fig. 4C). Our observations align with the results from a previous systematic analysis of hypermucoviscosity, which revealed the role of genes related to purine metabolism in the regulation of mucoviscosity (12).

In addition to genes related to metabolic pathways, other DEGs are involved in various cellular functions, such as transport and RNA binding. By combining GO enrichment analysis and DEG fold changes, we identified 24 potent hits (Table 2). These hits include 16 upregulated and eight downregulated genes, which were further investigated to evaluate their contributions to mucoviscosity.

**TABLE 2:** Genes differentially expressed between wild type (WT) and Δ*ompR* mutant

Description	Gene_id	Gene name	Description	TPM^*a*^		log2FC^*b*^	*P* value
				WT	Δ*ompR*		
Up-regulated	VK055_5116	*fyuA*	siderophore yersiniabactin receptor FyuA	2.27	75.1	4.89	6.55E-47
	VK055_3329	*atpI*	F0F1 ATP synthase subunit I	48.11	176.64	1.71	0.001
	VK055_3331	*atpE*	F0F1 ATP synthase subunit C	166.48	687.31	1.92	0.038
	VK055_3332	*atpF*	F0F1 ATP synthase subunit B	121.57	557.59	2.02	9.40E-18
	VK055_3333	*atpH*	F0F1 ATP synthase subunit delta	87.74	296.23	1.59	3.05E-09
	VK055_3334	*atpA*	F0F1 ATP synthase subunit alpha	101.13	434.34	1.93	2.14E-42
	VK055_3335	*atpG*	F0F1 ATP synthase subunit gamma	92.27	359.93	1.79	1.67E-24
	VK055_3336	*atpD*	F0F1 ATP synthase subunit beta	69.98	338.65	2.11	1.03E-39
	VK055_3337	*atpC*	F0F1 ATP synthase subunit epsilon	102.25	558.91	2.28	4.11E-16
	VK055_4142	*gcvT*	glycine cleavage system aminomethyltransferase GcvT	38.07	150.16	1.81	2.39E-17
	VK055_4143	*gcvH*	glycine cleavage system protein GcvH	140.99	643.30	2.03	2.31E-13
	VK055_4144	*gcvP*	aminomethyl-transferring glycine dehydrogenase	52.96	208.12	1.81	5.76E-41
	VK055_0304	*0304*	DUF1852 domain-containing protein	20.92	82.08	1.81	4.84E-10
	VK055_0305	*0305*	methionine synthase	41.13	153.21	1.73	9.42E-16
	VK055_3379	*3379*	PTS transporter subunit EIIC	4.93	26.94	2.28	4.35E-09
	VK055_3380	*3380*	6-phospho-alpha-glucosidase	11.15	99.92	2.99	3.15E-27
Down-regulated	VK055_4983	*4983*	mannitol dehydrogenase family protein	93.2	20.01	−2.39	4.94E-28
	VK055_4481	*4481*	ATP-binding cassette domain-containing protein	201.03	69.63	−1.70	2.62E-14
	VK055_4482	*4482*	metal ABC transporter permease	82.72	21.67	−2.1	1.49E-11
	VK055_4483	*4483*	metal ABC transporter substrate-binding protein	67.12	18.80	−2.00	4.13E-10
	VK055_1148	*pspC*	envelope stress response membrane protein PspC	221.79	73.53	−1.76	7.81E-05
	VK055_1149	*pspB*	envelope stress response membrane protein PspB	1233.65	320.17	−2.01	0.015
	VK055_1150	*pspA*	phage shock protein PspA	825.94	320.45	−1.53	7.92E-24
	VK055_3555	*3555*	MFS transporter	75.77	23.22	−1.87	2.95E-15

a TPM, transcripts per million reads.

b Fold change.

### OmpR regulons related to hypermucoviscosity.

**FIG 5:**
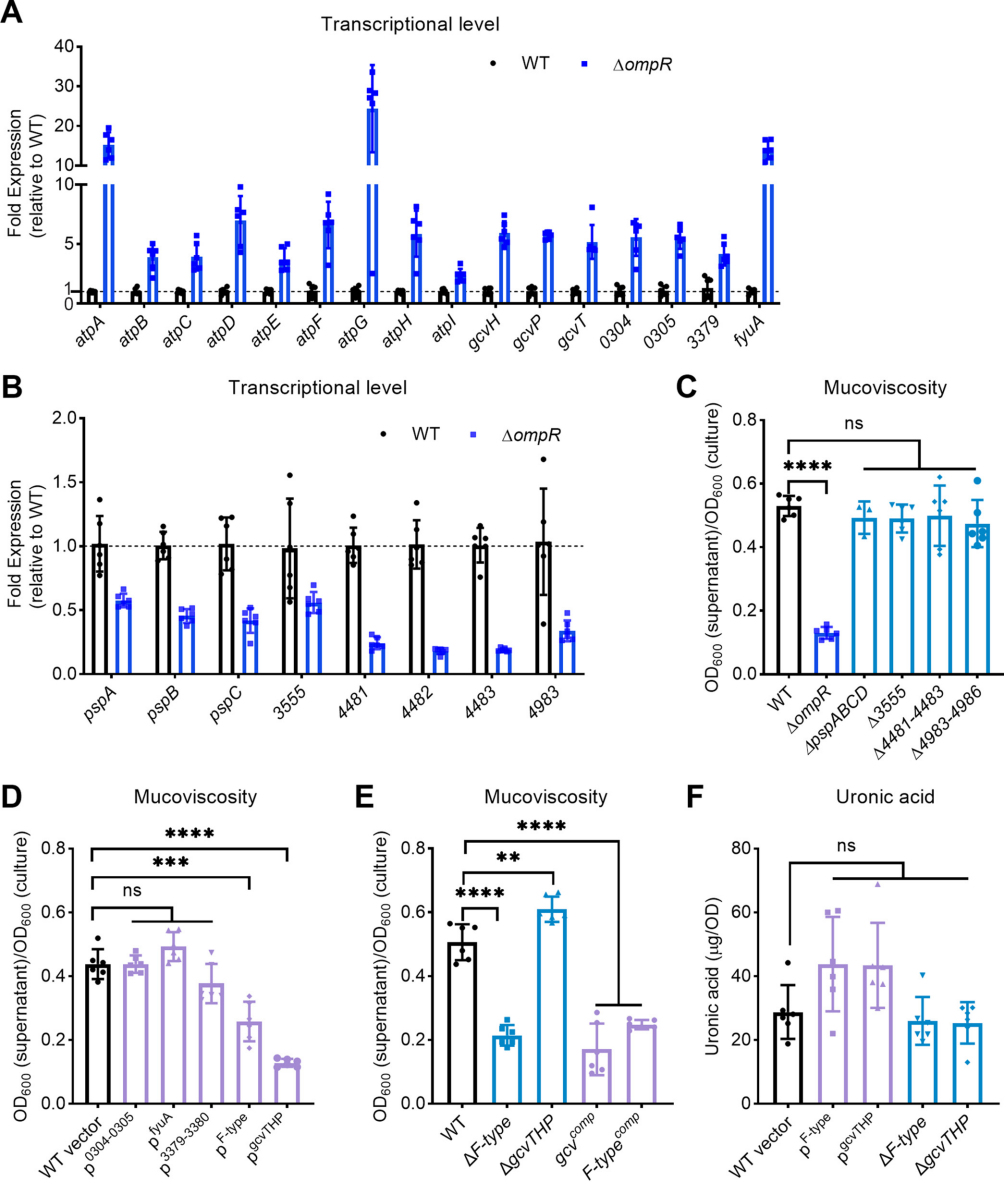
Overexpression of either *F-type atp* operon or *gcvTHP* reduces mucoviscosity. The relative expression of each upregulated gene (A) and downregulated gene (B) was validated by qRT-PCR. A dotted line denotes the mean value of wild type. Mucoviscosity was determined by sedimentation assays (C to E). Mutants for representative downregulated genes (C, E) and overexpression strains for representative upregulated genes (D) were constructed and evaluated for their mucoviscosity. Wild-type or wild-type strain carrying an empty vector (WT vector) was used as a control. (F) Uronic acid contents of wild-type and isogenic variants. Data are presented as mean ± SD from six biological replicates. A two-tailed *t* test was performed to determine the statistical significance of two group comparison. **, *P* < 0.01; ***, *P* < 0.001; ****, *P* < 0.0001; ns, no significance.

The transcriptomic results of significantly regulated genes (Table 2) were validated by qRT-PCR. The qRT-PCR data demonstrated the transcriptional levels of the *atp operon*, *gcvT*, *gcvH*, *gcvP*, *VK055*_0304-0305, *VK055*_3379, and *fyuA* were significantly upregulated (Fig. 5A). Similarly, the transcriptional levels of *psp*ABC and *VK055*_3555 were downregulated 2-fold, while those of *VK055*_4481-4483 and *VK055*_4983 were reduced approximately 5-fold (Fig. 5B), which is consistent with the transcriptomic results. Subsequently, we constructed corresponding isogenic mutants with markerless deletion or overexpression strains containing low-copy-number plasmid (Fig. S1) to evaluate whether they exhibited consistent viscosity phenotypes.

In the Δ*ompR* mutant, the expression of the following genes was repressed: *psp*ABCD, *VK055*_3555, *VK055*_4481-4483, and *VK055*_4983-4986. However, none of the deficient mutants altered the mucoviscosity compared to the wild-type strain (Fig. 5C). Nevertheless, overexpressing either *F-type atp operon* (p^F-type^, TH16249) or *gcvTHP* (p^gcvTHP^, TH16248) in *trans* in the wild-type strain led to reduced mucoidy (Fig. 5D). Although the expression of *VK055*_0305, a gene involved in the methionine metabolism supplying methyl group pool for DNA synthesis (Fig. 4C), increased approximately 8-fold in Δ*ompR* mutant (Fig. 5B), overexpressing of this gene in wild-type (p^0304-0305^, TH16246) did not affect mucoidy (Fig. 5D). Furthermore, we deleted the *F-type atp operon* and *gcvTHP* in the wild-type strain, respectively, to determine whether their deficiency would alter the mucoviscosity. The GcvTHP deficiency mutant Δ*gcvTHP* (TH16266) exhibited increased mucoviscosity (*P* < 0.01). Intriguingly, the F-type ATP synthase-deficiency mutant Δ*F-type* (TH16066) exhibited low mucoviscosity (Fig. 5E), likely due to a growth defect observed (Fig. S2). Moreover, complementation of the Δ*F-type* in *trans* (*F-type*^comp^, TH16941) produced a phenotype similar to overexpressing it in the wild-type strain background. Likewise, the complementation of Δ*gcvTHP* in *trans* (*gcv*^comp^, TH16942) resulted in a similar phenotype. Multiple copy numbers of the complementation plasmid used in the study are likely responsible for the observed overexpression phenotype (Fig. 5E).

Additionally, neither inactivation nor overexpression of *F-type atp operon* and *gcvTHP* genes altered the uronic acid content (Fig. 5F). These results suggest that F-type ATP synthase and GcvTHP are involved in the mucoviscosity formation, independent of the capsule level.

### Binding of recombinant OmpR to the promoter region of the *F-type atp operon*.

**FIG 6:**
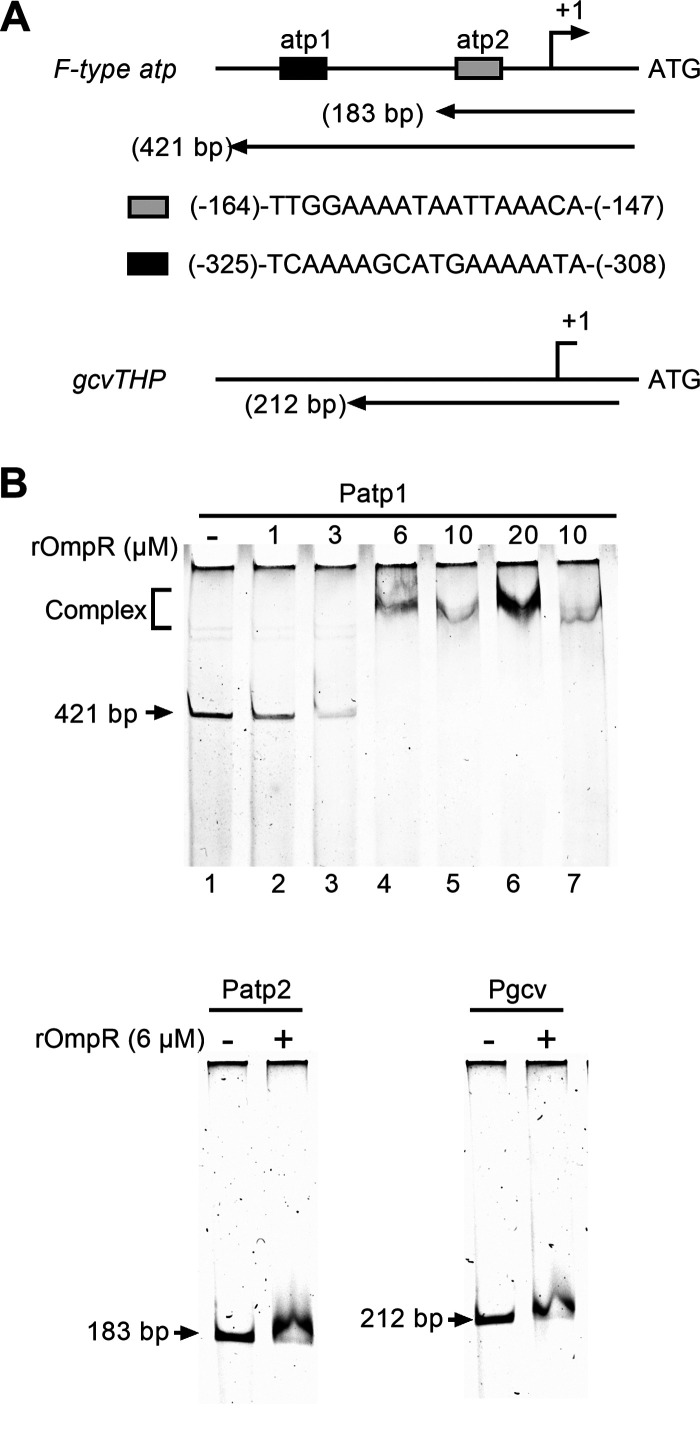
EMSA analysis of binding of recombinant OmpR to the *F-type atp* operon and *gcvTHP* promoter regions. (A) Diagrams of promoter regions of *F-type atp* operon and *gcvTHP* (not drawn to scale). Gray and black boxes represent two putative OmpR binding sequences, atp1 and atp2. Nucleotide numberings are relative to the *atpI* and *gcvT* ATG codons, respectively. The relative positions and lengths of the three DNA fragments used in EMSA are shown. (B) EMSA results of rOmpR. EMSA of Patp1 (Upper panel): the Patp1 DNA fragment was mixed with increasing concentrations of rOmpR-P (in the presence of acetyl phosphate) at 0, 1, 3, 6, 10, and 20 μM (Lanes 1 to 6) or 10 μM non-P rOmpR (in the absence of acetyl phosphate) (lane 7). EMSA of Patp2 and Pgcv (Lower panels) DNA fragments specified in the absence and presence of 6 μM rOmpR-P (in the presence of acetyl phosphate), respectively (−/+), were analyzed. DNA bands were detected by Gelred staining. The positions of DNA fragments not shifted were labeled.

As the expression of genes encoding F-type ATP synthase and GcvTHP was dramatically increased in the Δ*ompR* mutant, we hypothesized that OmpR might directly modulate these genes. To determine whether OmpR directly binds to their promoter regions, we conducted an electrophoretic mobility shift assay (EMSA). We searched the ATCC43816 genomic sequence (ID: CP009208) on an online gene regulation and gene expression database (https://www.prodoric.de/) using the OmpR matrix (MX000141 or MX000338) to identify putative binding sites (36). Two potential OmpR binding sites, (-164)-TTGGAAAATAATTAAACA-(-147) and (-325)-TCAAAAGCATGAAAAATA-(-308), were identified upstream of the *atpI* ATG codon, while no typical OmpR binding site was found upstream of the *gcvT* gene (Fig. 6A). In line with this, EMSA data suggest that recombinant OmpR (rOmpR) binds to the distant sequence upstream of *atpI* (Patp1) not the near sequence (Patp2) (Fig. 6B). Furthermore, unphosphorylated rOmpR directly bound to the promoter region of Patp1, which may explain why *ompR*^D55A^ and Δ*envZ* partially maintain mucoviscosity (Fig. 6B, lane 7). No binding activity was observed for the phosphorylated rOmpR with the promoter of gene *gcvT*. These results indicate that OmpR directly represses the expression of the *F-type atp* operon but indirectly represses the expression of the *gcvTHP* genes.

### High intracellular energy in low mucoviscosity isogenic strains.

**FIG 7:**
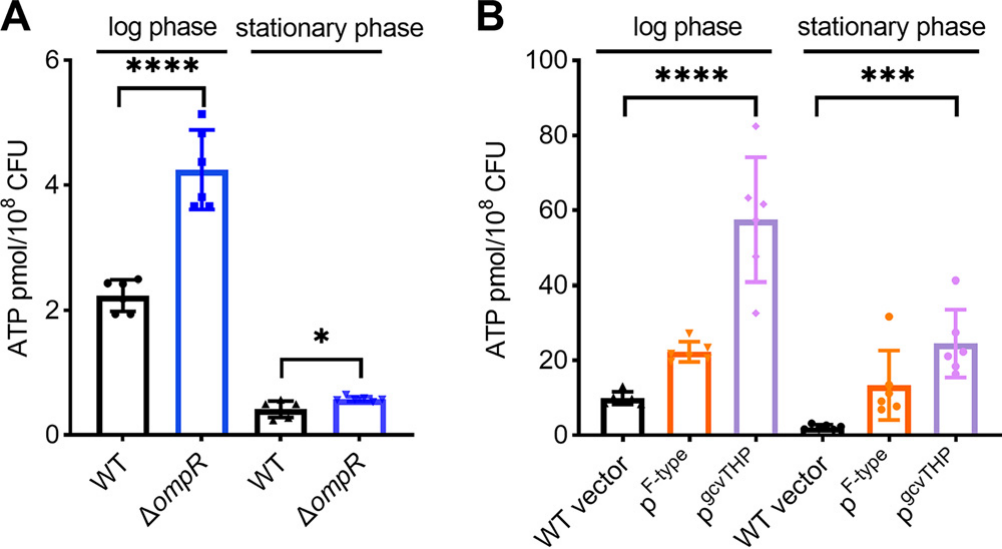
Intracellular ATP levels of cells at log phase and stationary phase. Intracellular ATP amounts determined in Δ*ompR* mutant (A) and overexpression of *F-type atp* operon or *gcvTHP* isogenic strains (B) were compared to the levels in wild-type cells. Data are presented as mean ± SD from six biological replicates. An *unpaired t test* was performed to determine the statistical significance of two-group comparisons and one-way ANOVA for multiple-group comparisons. *, *P* < 0.05; ***, *P* < 0.001; ****, *P* < 0.0001.

It is known that the central metabolism genes related to pyruvate metabolism and the TCA cycle influence hypermucoviscosity (12). We found, in our study, that the regulons of OmpR are associated with ATP or nucleic acid biosynthesis. Consequently, we hypothesized that intracellular energy levels play a role in reducing mucoviscosity. To test this hypothesis, we employed an ATP-dependent luciferase assay to measure the intracellular ATP levels as a proxy for cellular energy status. Our findings showed that the ATP level of log-phase Δ*ompR* cells was approximately twice as high as that of wild-type cells (4.25 versus 2.23 pmol/10^8^ CFU). Similarly, stationary-phase Δ*ompR* cells exhibited elevated ATP levels compared to wild-type cells (Fig. 7A). In accordance with these observations, we also found increased intracellular ATP levels in overexpression of either *F-type atp* operon or *gcvTHP* during both log phase and stationary phase (Fig. 7B). These results suggest that cellular energy status plays a role in modulating mucoviscosity. However, the precise mechanism by which elevated ATP production influences mucoviscosity remains unclear and warrants further investigation.

## DISCUSSION

Hypervirulent K. pneumoniae is known to cause severe infections (37). A key characteristic of HvKP is its highly stretchable colonies (38, 39). Capsule abundance (10) and elevated RmpD level (13) have been recognized as two major factors contributing to hypermucoviscosity. Although capsule production is required for hypermucoviscosity of K. pneumoniae, the mechanisms of RmpD-mediated hypermucoviscosity remain unknown. Furthermore, RmpD-negative hypermucoviscosity has been identified in numerous clinical strains (40–43). Therefore, the molecular event precisely modulating hypermucoviscosity formation is still unclear. Our current study systematically assessed the contributions of TCS response regulators to hypermucoviscosity and uncovered a metabolic association with OmpR-mediated hypermucoviscosity in K. pneumoniae.

Environmental factors are known to affect the hypermucoviscosity phenotype of K. pneumoniae (44, 45). As a result, K. pneumoniae may sense the environmental stimuli to regulate its mucoidy characteristic. Bacterial TCSs serve as critical sensors of these environmental cues. By systematic inactivation of genes coding for all 33 TCS response regulators in K. pneumoniae, we found that OmpR is essential for the hypermucoviscous trait in multiple clinical strains. Additionally, an Δ*ompR* mutant of K1 serotype strain NTUH-K2044 exhibited reduced mucoviscosity (46). Interestingly, only two response regulators, OmpR and RcsB, were required for hypermucoviscosity. While RcsB has been implicated in capsule regulation, affecting hypermucoviscosity, we investigated the potential cross-regulation between OmpR and RcsB due to the well-known interplay of TCSs in prokaryotes (47). Our results showed that OmpR deficiency did not affect the expression of all genes in the *rcsB* locus. Additionally, the expression of *rmpD* was also not altered in the Δ*ompR* mutant. These findings suggest that the OmpR response regulator independently regulates mucoviscosity. Although OmpR modulates mucoviscosity in various serotypes of K. pneumoniae strains, the molecular mechanisms underlying these regulatory processes have not been elucidated.

In this study, we determined that OmpR regulates mucoviscosity independently of the two well-documented determinants of hypermucoviscosity of K. pneumoniae, capsule and RmpD, indicating the involvement of a previously unknown mechanism in OmpR-mediated hypermucoviscosity. Transcriptomic analysis revealed that the absence of OmpR led to changes in the expression of a large number of genes in K. pneumoniae. Genes responsible for ATP biosynthesis and serine metabolism were the most differentially upregulated genes among 273 DEGs identified by enrichment analysis. The downregulation of genes encoding membrane proteins was also evident in the Δ*ompR* mutant. Only the *atp* operon, encoding F-type ATP synthase, and the *gcvTHP* encoding glycine cleavage system affected the mucoviscosity. Furthermore, OmpR directly bound the promoter region of the *atp* operon, not the *gcvTHP*, suggesting that OmpR directly regulates the expression of the *atp* operon while likely indirectly regulating *gcvTHP*, which requires further investigation.

F-type ATP synthase is a nanomotor in bacterial plasma membrane involved in ATP synthesis and hydrolysis. The direction of catalysis depends on the cellular bioenergetic conditions (48). ATP is a critical regulator of central metabolism, playing a crucial role in RNA, DNA, and protein synthesis (49). Similarly, the glycine cleavage system is a crucial enzyme complex of one-carbon metabolism, which catalyzes glycine into CO2, NH_4_^(+)^, and a methylene group. The methylene group is accepted by tetrahydrofolate (THF) to generate 5,10-methylenetetrahydrofolate, a precursor of amino acid and DNA synthesis. The glycine cleavage system is responsible for serine biosynthesis in Francisella tularensis and is required for its pathogenesis *in vivo* (50). Thus, both the F-type ATP synthase and the glycine cleavage system are closely related to cellular metabolism. In K. pneumoniae, cellular carbon metabolism has been shown to affect capsule biosynthesis via either glucose through the cAMP-dependent carbon catabolite repression (CCR) (51) or fucose-mediated expression of the *rmpD* operon (52). This study demonstrated that capsule levels remain unchanged in Δ*ompR* and variants of overexpression of either *F-type atp* operon or *gcvTHP*. Therefore, the precise link between the F-type ATP synthase and glycine cleavage system to mucoviscosity remains unknown.

As F-type ATP synthase is directly related to cellular energy conditions, the regulatory mechanism appears to be connected to energy metabolism. The Δ*ompR* mutant significantly increases ATP production. The overexpression of either F-type ATP synthase or GcvTHP enhanced ATP production, which may increase the ATP/ADP ratio. A high cytosolic ATP/ADP ratio inhibits glycolysis, while a lower cytosolic ATP/ADP ratio enhances glycolysis (53). Consequently, high ATP levels in Δ*ompR* cells could inhibit glycolysis, leading to the high production of secondary lactic acid or carboxylic acid. These acids influence the intracellular pH, which drives the switch from a proton-driven ATP synthase to a proton-pumping ATPase (54). Alongside the proton-pumping activity, relatively low extracellular pH appears to promote the breakdown of the glycosidic bonds of polysaccharides to reduce mucoviscosity. We also noted that the Δ*F-type* mutant also exhibited reduced mucoviscosity, indicating the important role of energy homeostasis in mucoviscosity. Notably, overexpression of either F-type ATP synthase or GcvTHP modestly inhibited the mucoviscosity compared with the Δ*ompR* mutant, suggesting genes or gene clusters other than *atp operon* and *gcvTHP* also contribute to hypermucoviscosity formation. The precise modulation of mucoviscosity by energy metabolism alteration due to the OmpR deficiency awaits further investigation.

In conclusion, OmpR is required for hypermucoviscosity and virulence of K. pneumoniae. OmpR regulates energy metabolism, thereby reducing mucoviscosity. This new insight into the link between OmpR-mediated bacterial energy metabolism and hypermucoviscosity enhances our understanding of the complex relationship between bacterial metabolism and mucoviscosity.

## MATERIALS AND METHODS

### Bacterial strains and growth conditions.

All bacterial isolates and derivatives of K. pneumoniae ATCC43816 are listed in Table S1. K. pneumoniae strains were grown in Luria-Bertani (LB) (Miller) broth or on Columbia blood plate (BAP) (Oxoid). The following antibiotics or chemicals were added as specified: apramycin (30 μg/mL), spectinomycin (300 μg/mL), L-arabinose (0.2%), kanamycin (50 μg/mL), and sucrose (5%).

### String test and sedimentation assay used to determine mucoviscosity.

The hypermucoviscosity was assessed by a positive string test and a semiquantitative sedimentation assay. The formation of >5-mm viscous string indicates a positive string test when a loop was used to lift a portion of a colony from a fresh colony of K. pneumoniae on BAP (55). In addition, the sedimentation assay was conducted as previously described (46). Briefly, K. pneumoniae was grown overnight in a 6-mL LB medium at 37°C with shaking at 180 rpm. The culture's optical density at 600 nm (OD_600nm_) was measured by spectrophotometry. The culture was sedimented at 1,000 g for 10 min unless otherwise mentioned. The OD_600nm_ ratio of the supernatant to the culture before centrifugation indicated the mucoviscosity semiquantitatively. Sedimentation values were used to classify mucoviscosity: hypermucoviscosity, >0.4; low viscosity, 0.2 to 0.4; and no viscosity, <0.2.

### Anthony’s capsule stain and capsule quantification.

Capsules of K. pneumoniae grown on BAP were stained following Anthony’s staining protocol (56) and imaged at ×1,000 magnification. The minor axis of the pale blue zone surrounding 20 random cells was measured by Image J to quantify capsule thickness.

Quantification of the capsule was performed as previously described (57). In brief, bacterial culture grown overnight in LB was mixed with 1% Zwittergent 3-14 detergent (Sigma-Aldrich) in 100 mM citric acid (pH 2.0) and incubated at 50°C for 20 min. The supernatant was precipitated with absolute ethanol. The resulting pellet was dried and resuspended in distilled water. Subsequently, 12.5 mM borax (Sigma-Aldrich) in H_2_SO_4_ was added to samples and boiled followed by addition of 0.15% 3-hydroxydiphenol (Sigma-Aldrich), and the absorbance at 520 nm was measured. The uronic acid content was determined from a standard curve of glucuronic acid (Sigma-Aldrich) and presented as micrograms per OD_600nm_.

### Construction of in-frame deletion mutants and site-directed mutagenesis.

All isogenic strains in K. pneumoniae, including markerless deletion mutants, point mutations, and complementation, were generated by a CRISPR-Cas9-mediated genome-editing method (25) and listed in Table S2. Briefly, designed sgRNAs were cloned onto the pSGKP plasmid, and dsDNA homologous arms of genes of interest were amplified by fusion PCR using PrimeSTAR DNA polymerase (TaKaRa). Plasmid pSGKP-sgRNA and its matched dsDNA homologous arms were electro-transformed into pCasKP-harboring K. pneumoniae competent cells cultured in LB supplemented with 0.2% L-arabinose. Transformants were selected with apramycin and spectinomycin at 30°C and verified by DNA sequencing. Finally, the plasmids were cured with 5% sucrose at 37°C. The plasmids and primers used in mutagenesis are listed in Table S3 and S4, respectively.

### Overexpression of genes.

Plasmid pACYC184-spe or pIB166-spe was constructed by replacing the chloramphenicol resistance gene of the pACYC184 with the spectinomycin resistance gene (spe). The promoter region of *rpsL* in K. pneumoniae ATCC43816 was cloned into pACYC184-spe and designated pTH16235. Strains that overexpressed genes of choice were generated by cloning the genes of interest into the pTH16235 vector by NEBuilder HiFi DNA Assembly Cloning Kit (New England BioLabs, USA). Briefly, the open reading frame and the plasmid backbone were amplified with primers listed in Table S4. The gel-purified PCR products were then assembled at 50°C for 30 min according to the manufacturer’s instructions. Next, the assembled products were directly transferred into E. coli DH5α to amplify plasmids overexpressing the genes of interest. Finally, the overexpression plasmids were electro-transformed into K. pneumoniae competent cells to generate corresponding overexpression strains.

### RNA-seq analysis.

Total RNA was extracted from a culture grown on BAP overnight using RNAprep pure Cell/Bacteria Kit (Tiangen BioTech, China) according to the manufacturer’s instructions and further purified with an RNeasy minikit (Qiagen, Germany). RNA-seq was performed at the Novogene Bioinformatics Technology (Beijing, China). Trimmed reads were mapped to the genome of K. pneumoniae ATCC43816 (CP009208) using Bowtie 2.3.1 and Tophat 2.1.1. The result of each sample represents the means of two biological replicates. All the bioinformatics analyses were performed using the free online platform of Majorbio Cloud Platform (www.majorbio.com) from Shanghai Majorbio Bio-pharm Technology Co., Ltd. In brief, the RSEM software was used to quantify the gene, and its isoform abundances from paired-end RNA-Seq data, the calculated TPMs (transcripts per million reads) were then directly used for comparing the differences in gene expression among samples using the DESeq2 software, and the corrected *P*-values (false discovery rate, FDR) were calculated with Benjamini/Hochberg’s method. Subsequently, the DEGs were identified based on |log2FC| ≥1, FDR < 0.05, and TPM > 20. Finally, GO enrichment of the DEGs was carried out to identify the statistically significant differences in functional and biological metabolic pathways levels using the Goatools (https://github.com/tanghaibao/GOatools) and Pathway Tools 26.0 (34), respectively.

### Quantitative RT-PCR.

Total RNA was extracted from bacterial cultures grown on BAP overnight using RNAprep pure Cell/Bacteria Kit (Tiangen Biotech, China) according to the manufacturer’s instructions. cDNA was produced using ReverTra Ace qPCR RT Master Mix with gDNA Remover (TOYOBO Biotech, China). qRT-PCR was conducted using UltraSYBR Mixture (CWBIO, China) with the Bio-Rad CFX96 system. The gene expression was normalized to 16S rRNA transcript abundance. The relative difference in mRNA levels was calculated using the 2^-ΔΔCt^ method (58). Two independent experiments with three technical repeats were performed for each qRT-PCR analysis.

### Quantitation of intracellular ATP.

BacTiter-Glo Microbial Cell Viability Assay (Promega, USA) was utilized to determine the intracellular ATP level. Bacteria were grown in LB Broth (Miller) at 37°C overnight. The overnight culture was diluted in fresh LB Broth and incubated at 37°C with shaking at 180 rpm. Samples were taken at log-phase grown cultures (OD_600nm_ = 0.6) and measured by the BacTiter-Glo Assay. Luminescence was recorded on a luminometer (BioTek Synergy H1). The CFU were determined in parallel and used to calculate the ATP amount per 10^8^ CFU.

### Luciferase reporter assay.

The luciferase reporter of *rmpADC* was constructed as follows: the promoter region of *rmp*ADC was cloned into the upstream of the promoterless luciferase gene in pTH15802 using NEBuilder HiFi DNA Assembly Cloning Kit (New England BioLabs). The resultant plasmid was transformed into competent cells of K. pneumoniae. The reporter strain was confirmed by PCR and DNA sequencing. Relative luminescence units (RLU) were measured by a luminometer (BioTek Synergy H1) and normalized to bacterial OD_600nm_.

### Purification of recombinant OmpR.

The C-terminally His-tagged recombinant OmpR (rOmpR) protein was constructed and purified. In brief, the full-length OmpR coding open reading frame was amplified with a primer pair Pr16788/Pr16789. The PCR product was ligated to the pET28a vector (Novagen) and transformed into E. coli BL21(DE3). E. coli strain BL21(DE3) carrying rOmpR (TH14685) grown to OD_600nm_ = 0.6 at 37°C was induced with 0.5 mM isopropyl β-D-1-thiogalactopyranoside (IPTG) at 18°C for 16 h in LB. Protein rOmpR was purified from the harvested cell pellets using nickel-nitrilotriacetic acid (Ni-NTA) affinity resin (Sangon Biotech, Shanghai, China) according to the manufacturer’s instructions. The purity of rOmpR was >95%, as determined by Coomassie blue-stained SDS polyacrylamide gel. Protein rOmpR was dialyzed overnight with a buffer with 20 mM Tris, 500 mM NaCl, 5% glycerol, pH 8.0.

### Electrophoretic mobility shift assay.

Electrophorectic mobility shift assays (EMSAs) were performed as previously described (59) using an EMSA kit (Viagene Biotech Inc., USA) and modified. Promoter regions of *atpI* and *gcvT* in K. pneumoniae ATCC43816 were amplified using respective primer sets. Purified OmpR-6×His from 0 to 20 μM mixed with 40 ng of amplified DNA fragments, along with 25 mM acetyl phosphate, 1 mM MgCl_2_, and 1 μL of poly(dI-dC), were incubated for 20 min at room temperature, and the reaction mixtures were stopped by adding loading buffer. The mixtures were separated by electrophoresis on 6% nondenaturing acrylamide gel running in 0.25× Tris Borate EDTA (TBE) buffer and stained by Gelred staining for bound DNAs.

### Murine pneumonia model.

The murine pneumonia model was used to evaluate virulence. Female CD1 mice (6 to 8 weeks old) were intranasally inoculated with approximately 2,000 CFU of log-phase grown K. pneumoniae per mouse. The virulence of strains was evaluated using a survival curve (*n* = 5). Because hvKP can disseminate to multiple organs, bacterial colonization was assessed by numerating bacteria numbers in multiple organs, including the lung, liver, and spleen, at 72 h postinfection. The data are presented as CFU/gram tissue (*n* = 3). The experiments were repeated twice. The study was approved by the Institutional Animal Care and Use Committee at Tsinghua University in Beijing.

### Statistical analysis.

The statistical analyses were conducted with the GraphPad Prism software 9.3.1, and the relevant data are presented as mean ± SD unless stated otherwise. Significant differences are defined by *P* values of < 0.05 (*), <0.01 (**), <0.001 (***), and < 0.0001 (****). Statistical analysis for each figure is denoted in the figure legends.

### Data availability.

The raw RNA-seq data presented in this study are available in NCBI’s Gene Expression Omnibus (GEO) database (accession GSE197039).
