# Supplementary material for: Two-Component Response Regulator OmpR Regulates Mucoviscosity through Energy Metabolism in Klebsiella pneumoniae
Source: Microbiol Spectr. 2023 Apr 25;11(3):e00544-23. doi: 10.1128/spectrum.00544-23 (PMC10269446; doi:10.1128/spectrum.00544-23)
Supplement: Supplemental file 1 — Fig. S1 and S2 and Tables S1 to S4. Download spectrum.00544-23-s0001.pdf, PDF file, 1.2 MB [file spectrum.00544-23-s0001.pdf]

SUPPORTING INFORMATION

Figure S1. Overexpression of target genes.

Figure S2. Growth curve of various *K. pneumoniae* strains.

Table S1. Laboratory strains and derivatives in this study.

Table S2. Constructions of strains used in this study.

Table S3. Plasmids used in this study.

Table S4. Primers used in this study.

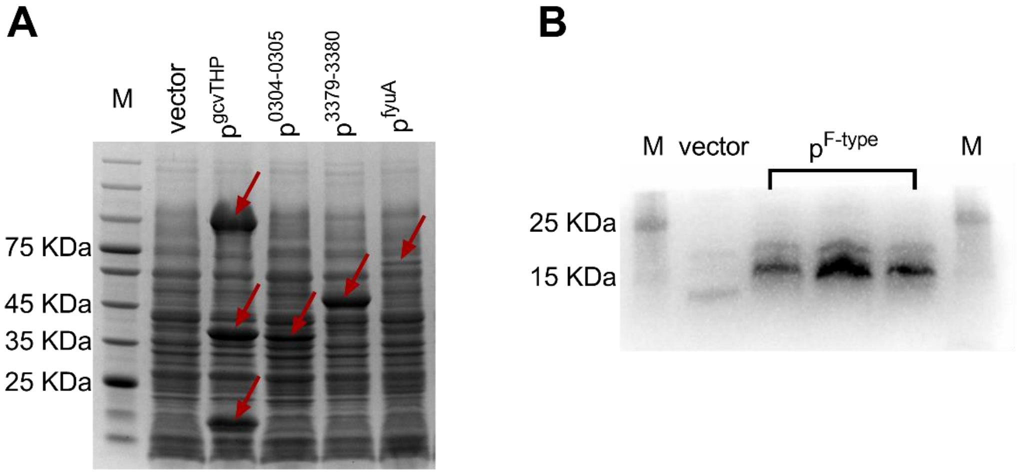

Figure S1. Overexpression of target genes. (A) 4-20% SDS-PAGE images of total proteins of strains cultured in LB broth and stained by Coomassie brilliant blue. The respectively overexpressed proteins were denoted with red arrows. (B) Western blot image of His-tagged AtpC in WTp$^{F\text{-}type}$.

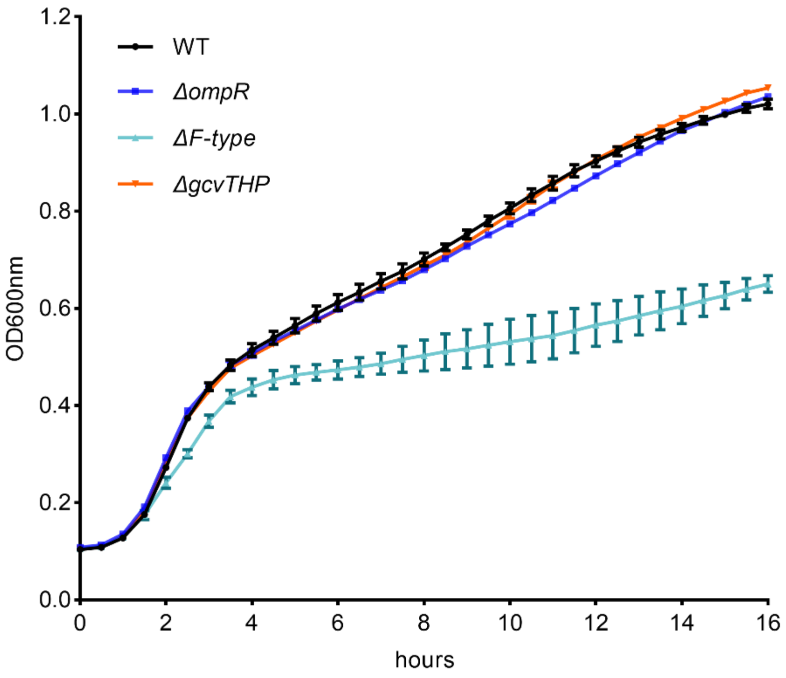

Figure S2. Growth curve of various *K. pneumoniae* strains. Overnight culture of each strain (Wild-type WT, Δ*ompR*, Δ*F-type*, and Δ*gcvTHP*) was diluted to 0.1 of OD_{600nm} in fresh LB medium. Then aliquots (200 µl) of diluted culture were added to the 96-well plate to culture at 37°C. The OD_{600nm} was monitored every 30 min by a microplate reader (BioTek Synergy H1). Data are presented as mean ± SD from three biological replicates.

Table S1. Laboratory strains and derivatives in this study

Strain ID	Strain name	Description	Reference or source
ATCC43816	ATCC43816	*Klebsiella pneumoniae*, serotype K2	ATCC
TH12887	TH12887	*K. pneumoniae*, clinical strain, serotype K2	This study
TH13044	TH13044	*K. pneumoniae*, clinical strain, serotype K2	This study
TH12896	TH12896	*K. pneumoniae*, clinical strain, serotype K23	This study
DH5α		*Escherichia coli* DH5α	NEB
BL21(DE3)		*E. coli* BL21(DE3)	NEB
TH13179		*K. pneumoniae* ATCC43816 derivative, carrying pCasKP, Apr^R	This study
TH14397		ATCC43816, Δ*VK055*_0032	This study
TH14423		ATCC43816, Δ*VK055*_0233	This study
TH14429		ATCC43816, Δ*VK055*_0249	This study
TH14411		ATCC43816, Δ*VK055*_0682	This study
TH14412		ATCC43816, Δ*VK055*_0953	This study
TH14413		ATCC43816, Δ*VK055*_0985	This study
TH14415		ATCC43816, Δ*VK055*_1326	This study
TH14416		ATCC43816, Δ*VK055*_1598	This study
TH14428		ATCC43816, Δ*VK055*_1724	This study
TH14417		ATCC43816, Δ*VK055*_1819	This study
TH14424		ATCC43816, Δ*VK055*_2216	This study
TH14398		ATCC43816, Δ*VK055*_2521	This study
TH16060		ATCC43816, Δ*VK055*_2547	This study
TH14426		ATCC43816, Δ*VK055*_2575	This study
TH14418		ATCC43816, Δ*VK055*_2578	This study
TH14419		ATCC43816, Δ*VK055*_2704	This study
TH14402		ATCC43816, Δ*VK055*_2984	This study
TH14401		ATCC43816, Δ*VK055*_3087	This study
TH14420		ATCC43816, Δ*VK055*_3257	This study
TH14406		ATCC43816, Δ*VK055*_3297	This study
TH14421		ATCC43816, Δ*VK055*_3393	This study
TH14427	Δ*ompR*	ATCC43816, Δ*VK055*_3696	This study
TH14410		ATCC43816, Δ*VK055*_3990	This study
TH14400		ATCC43816, Δ*VK055*_4034	This study
TH16062		ATCC43816, Δ*VK055*_4175	This study
TH14425		ATCC43816, Δ*VK055*_4178	This study
TH16064		ATCC43816, Δ*VK055*_4202	This study
TH14409		ATCC43816, Δ*VK055*_4622	This study
TH14422		ATCC43816, Δ*VK055*_4775	This study
TH14399	Δ*rcsB*	ATCC43816, Δ*VK055*_4883	This study
TH14408		ATCC43816, Δ*VK055*_4961	This study
TH14404		ATCC43816, Δ*VK055*_4995	This study
TH14403		ATCC43816, Δ*VK055*_5004	This study
TH14470		ATCC43816, Δ*ompR*, carrying pCasKP, Apr^R	This study
TH14438	$ompR^{comp}$	ATCC43816, Δ*ompR*::*ompR*	This study
TH14495	$ompR^{D55A}$	ATCC43816, Δ*ompR*::$ompR^{D55A}$	This study

TH16268		TH12887, Δ*ompR*	This study
TH16270		TH13044, Δ*ompR*	This study
TH16070		TH12896, Δ*ompR*	This study
TH14405	Δ*wzab*	ATCC43816, Δ*wzab*	This study
TH14889	Δ*envZ*	ATCC43816, Δ*envZ*	This study
TH15057	Δ4983-4986	ATCC43816, Δ*VK055*_4983-4986	This study
TH15395	Δ4481-4483	ATCC43816, Δ*VK055*_4481-4483	This study
TH15092	Δ3555	ATCC43816, Δ*VK055*_3555	This study
TH16173	Δ*pspABCD*	ATCC43816, Δ*pspABCD*	This study
TH16266	Δ*gcvTHP*	ATCC43816, Δ*gcvTHP*	This study
TH16942	*gcv*^{comp}	Δ*gcvTHP,* carrying pACYC184*ΔcatΔtet*::*gcvTHP*, Spe^R	This study
TH16066	Δ*F-type*	ATCC43816, Δ*atpIBEFHAGDC*	This study
TH16941	*F-type*^{comp}	Δ*F-type,* carrying pACYC184*ΔcatΔtet*::*atpIBEFHAGDC*, Spe^R	This study
TH16244	WT vector	ATCC43816, carrying pACYC184*ΔcatΔtet*::P*rpsL*, Spe^R	This study
TH16245	p$^{3379\text{-}3380}$	ATCC43816, carrying pACYC184*ΔcatΔtet*::*VK055*_3379-3380, Spe^R	This study
TH16246	p$^{0304\text{-}0305}$	ATCC43816, carrying pACYC184*ΔcatΔtet*::*VK055*_0304-0305, Spe^R	This study
TH16247	p^{fyuA}	ATCC43816, carrying pACYC184*ΔcatΔtet*::*fyuA*, Spe^R	This study
TH16248	p^{gcvTHP}	ATCC43816, carrying pACYC184*ΔcatΔtet*::*gcvTHP*, Spe^R	This study
TH16249	p$^{F\text{-}type}$	ATCC43816, carrying pACYC184*ΔcatΔtet*::*atpIBEFHAGDC*, Spe^R	This study
TH15777		ATCC43816, carrying pACYC184*Δcat*::luciferase, Spe^R	This study
TH15778		ATCC43816 *ΔompR*, carrying pACYC184*Δcat*::luciferase, Spe^R	This study
TH15814		ATCC43816, carrying pACYC184*Δcat*::P*rmpADC*-luciferase, Spe^R	This study
TH15815		ATCC43816 *ΔompR*, carrying pACYC184*Δcat*::P*rmpADC*-luciferase, Spe^R	This study
TH14683		*E. coli* DH5α, carrying pET28a::*ompR*, Kan^R	This study
TH14685		*E. coli* BL21, carrying pET28a::*ompR*, Kan^R	This study

Spe^R: spectinomycin resistance; Apr^R: apramycin resistance; Kan^R: kanamycin resistance; ATCC: American Type Culture Collection; NEB: New England Biolabs LTD.

Table S2. Constructions of strains used in this study

Strain ID	Genotype	Donor DNA used for mutagenesis		Template DNA	Recipient or parental strain
		Plasmid	Repair template		
TH13179	ATCC43816; pCasKP-apr	pCasKP-apr	N/A	N/A	ATCC43816
TH14397	Δ*VK055*_0032	pTH14355	Pr16207/16208 Pr16209/16210	ATCC43816	TH13179
TH14423	Δ*VK055*_0233	pTH14381	Pr16505/16506 Pr16507/16508	ATCC43816	TH13179
TH14429	Δ*VK055*_0249	pTH14387	Pr16402/16403 Pr16404/16405	ATCC43816	TH13179
TH14411	Δ*VK055*_0682	pTH14369	Pr16212/16213 Pr16214/16215	ATCC43816	TH13179
TH14412	Δ*VK055*_0953	pTH14370	Pr16217/16218 Pr16219/16220	ATCC43816	TH13179
TH14413	Δ*VK055*_0985	pTH14371	Pr16222/16223 Pr16224/16225	ATCC43816	TH13179
TH14415	Δ*VK055*_1326	pTH14373	Pr16232/16233 Pr16234/16235	ATCC43816	TH13179
TH14416	Δ*VK055*_1598	pTH14374	Pr16237/16238 Pr16239/16240	ATCC43816	TH13179
TH14428	Δ*VK055*_1724	pTH14386	Pr16372/16373 Pr16374/16375	ATCC43816	TH13179
TH14417	Δ*VK055*_1819	pTH14375	Pr16242/16243 Pr16244/16245	ATCC43816	TH13179
TH14424	Δ*VK055*_2216	pTH14382	Pr16542/16543 Pr16544/16545	ATCC43816	TH13179
TH14398	Δ*VK055*_2521	pTH14356	Pr16387/16388 Pr16389/16390	ATCC43816	TH13179
TH16060	Δ*VK055*_2547	pTH16040	Pr18260/18261 Pr18262/18263	ATCC43816	TH13179
TH14426	Δ*VK055*_2575	pTH14384	Pr16354/16355 Pr16356/16357	ATCC43816	TH13179
TH14418	Δ*VK055*_2578	pTH14376	Pr16314/16315 Pr16316/16317	ATCC43816	TH13179
TH14419	Δ*VK055*_2704	pTH14377	Pr16377/16378 Pr16379/16380	ATCC43816	TH13179
TH14402	Δ*VK055*_2984	pTH14360	Pr16319/16320 Pr16321/16322	ATCC43816	TH13179
TH14401	Δ*VK055*_3087	pTH14359	Pr16324/16325 Pr16326/16327	ATCC43816	TH13179
TH14420	Δ*VK055*_3257	pTH14378	Pr16382/16383 Pr16384/16385	ATCC43816	TH13179
TH14406	Δ*VK055*_3297	pTH14364	Pr16510/16511 Pr16512/16513	ATCC43816	TH13179
TH14421	Δ*VK055*_3393	pTH14379	Pr16527/16528 Pr16529/16530	ATCC43816	TH13179

TH14427	Δ*ompR*	pTH14385	Pr16520/16521 Pr16522/16523	ATCC43816	TH13179
TH14410	Δ*VK055*_3990	pTH14368	Pr16392/16393 Pr16394/16395	ATCC43816	TH13179
TH14400	Δ*VK055*_4034	pTH14358	Pr16329/16330 Pr16331/16332	ATCC43816	TH13179
TH16062	Δ*VK055*_4175	pTH16053	Pr18264/18265 Pr18266/18267	ATCC43816	TH13179
TH14425	Δ*VK055*_4178	pTH14383	Pr16495/16496 Pr16497/16498	ATCC43816	TH13179
TH16064	Δ*VK055*_4202	pTH16042	Pr18268/18269 Pr18270/18271	ATCC43816	TH13179
TH14409	Δ*VK055*_4622	pTH14367	Pr16537/16538 Pr16539/16540	ATCC43816	TH13179
TH14422	Δ*VK055*_4775	pTH14380	Pr16500/16501 Pr16502/16503	ATCC43816	TH13179
TH14399	Δ*rcsB*	pTH14357	Pr16547/16548 Pr16549/16550	ATCC43816	TH13179
TH14408	Δ*VK055*_4961	pTH14366	Pr16334/16335 Pr16336/16337	ATCC43816	TH13179
TH14404	Δ*VK055*_4995	pTH14362	Pr16339/16340 Pr16341/16342	ATCC43816	TH13179
TH14403	Δ*VK055*_5004	pTH14361	Pr16344/16345 Pr16346/16347	ATCC43816	TH13179
TH14405	Δ*wzab*	pTH14363	Pr16532/16533 Pr16534/16535	ATCC43816	TH13179
TH14438	*ompR*^{comp}	pTH14396	Pr16520/16523	ATCC43816	TH14470
TH14495	*ompR*^{D55A}	pTH14396	Pr16520/16516 Pr16517/16515	ATCC43816	TH14470
TH16268	TH12887 *ΔompR*	pTH14385	Pr16520/16521 Pr16522/16523	TH12887	TH12887 pCasKP-apr
TH16270	TH13044 *ΔompR*	pTH14385	Pr16520/16521 Pr16522/16523	TH13044	TH13044 pCasKP-apr
TH16070	TH12896 Δ*ompR*	pTH14385	Pr16520/16521 Pr16522/16523	TH12896	TH12896 pCasKP-apr
TH14889	*ΔenvZ*	pTH14807	Pr17080/17081 Pr17082/17083	ATCC43816	TH13179
TH15057	*Δ4983*	pTH15056	Pr17242/17243 Pr17244/17245	ATCC43816	TH13179
TH15395	*Δ4481-4483*	pTH15304	Pr17419/17420 Pr17421/17422	ATCC43816	TH13179
TH15092	*Δ3555*	pTH15094	Pr17256/17257 Pr17258/17259	ATCC43816	TH13179
TH16066	*ΔF-type*	pTH15451	Pr18282/18283 Pr18284/18285	ATCC43816	TH13179
TH16266	*ΔgcvTHP*	pTH16265	Pr18490/18491 Pr18492/18493	ATCC43816	TH13179

TH16173	*ΔpspABCD*	pTH16138	Pr18365/18366<br>Pr18367/18368	ATCC43816	TH13179
TH16244	WT vector	pTH16235	N/A	N/A	ATCC43816
TH16245	WT p3379-3380	pTH16236	N/A	N/A	ATCC43816
TH16246	WT p0304-0305	pTH16237	N/A	N/A	ATCC43816
TH16247	WT p*fyuA*	pTH16238	N/A	N/A	ATCC43816
TH16248	WT p*gcvTHP*	pTH16239	N/A	N/A	ATCC43816
TH16249	WT pF-type	pTH16240	N/A	N/A	ATCC43816
TH16941	*F-type*^{comp}	pTH16240	N/A	N/A	TH16066
TH16942	*gcv*^{comp}	pTH16239	N/A	N/A	TH16266
TH15777	ATCC43816 derivative, carrying pACYC184*Δcat*::luciferase	pTH15802	N/A	N/A	ATCC43816
TH15778	△ompR derivative, carrying pACYC184*Δcat*::luciferase	pTH15802	N/A	N/A	TH14427
TH15814	ATCC43816 derivative, carrying pACYC184*Δcat*::P*rmpADC*-luciferase	pTH15807	N/A	N/A	ATCC43816
TH15815	ΔompR derivative, carrying pACYC184*Δcat*::P*rmpADC*-luciferase	pTH15807	N/A	N/A	TH14427

Table S3. Plasmids used in this study

Plasmid	Descriptions	Reference or source
pCasKP	Thermosensitive plasmid carrying *Cas9*, ParaC, ParaB, Apr^R	(1)
pSGKP	Expressing sgRNA and carrying *sacB*, Spe^R	(1)
pACYC184	Cm^R, Tc^R	ATCC
pET-28a	N-His, N-Thrombin, N-T7, C-His, Kan^R	Novagen
pTH15652	pACYC184 derivative, *Δcat*, C-His, Spe^R	This study
pTH15802	pTH15652 derivative, pACYC184*Δcat*::luciferase, Spe^R	This study
pTH15807	pTH15802 derivative, pACYC184*Δcat*::P*rmpADC*-luciferase, Spe^R	This study
pTH15493	pIB166 derivative, carrying pIB166::*atpIBEFHAGDC*, Spe^R	This study
pTH16235	pTH15652 derivative, carrying Promoter of rpsL, C-His, Spe^R	This study
pTH16236	pTH16235 derivative, carrying pACYC184*ΔcatΔtet*::*VK055*_3379-3380, Spe^R	This study
pTH16237	pTH16235 derivative, carrying pACYC184*ΔcatΔtet*::*VK055*_0304-0305, Spe^R	This study
pTH16238	pTH16235 derivative, carrying pACYC184*ΔcatΔtet*::*fyuA*, Spe^R	This study
pTH16239	pTH16235 derivative, pACYC184*ΔcatΔtet*::*gcvTHP*, Spe^R	This study
pTH14355	pSGKP with *VK055*_0032 spacer, Spe^R	This study
pTH14381	pSGKP with *VK055*_0233 spacer, Spe^R	This study
pTH14387	pSGKP with *VK055*_0249 spacer, Spe^R	This study
pTH14369	pSGKP with *VK055*_0682 spacer, Spe^R	This study
pTH14370	pSGKP with *VK055*_0953 spacer, Spe^R	This study
pTH14371	pSGKP with *VK055*_0958 spacer, Spe^R	This study
pTH14373	pSGKP with *VK055*_1326 spacer, Spe^R	This study
pTH14374	pSGKP with *VK055*_1598 spacer, Spe^R	This study
pTH14386	pSGKP with *VK055*_1724 spacer, Spe^R	This study
pTH14375	pSGKP with *VK055*_1819 spacer, Spe^R	This study
pTH14382	pSGKP with *VK055*_2216 spacer, Spe^R	This study
pTH14356	pSGKP with *VK055*_2521 spacer, Spe^R	This study
pTH16040	pSGKP with *VK055*_2547 spacer, Spe^R	This study
pTH14384	pSGKP with *VK055*_2575 spacer, Spe^R	This study
pTH14376	pSGKP with *VK055*_2578 spacer, Spe^R	This study
pTH14377	pSGKP with *VK055*_2704 spacer, Spe^R	This study
pTH14360	pSGKP with *VK055*_2984 spacer, Spe^R	This study
pTH14359	pSGKP with *VK055*_3087 spacer, Spe^R	This study
pTH14378	pSGKP with *VK055*_3257 spacer, Spe^R	This study
pTH14364	pSGKP with *VK055*_3297 spacer, Spe^R	This study
pTH14379	pSGKP with *VK055*_3393 spacer, Spe^R	This study
pTH14385	pSGKP with *ompR* spacer, Spe^R	This study
pTH14368	pSGKP with *VK055*_3990 spacer, Spe^R	This study
pTH14358	pSGKP with *VK055*_4034 spacer, Spe^R	This study
pTH16053	pSGKP with *VK055*_4175 spacer, Spe^R	This study
pTH14383	pSGKP with *VK055*_4178 spacer, Spe^R	This study
pTH16042	pSGKP with *VK055*_4202 spacer, Spe^R	This study
pTH14367	pSGKP with *VK055*_4622 spacer, Spe^R	This study
pTH14380	pSGKP with *VK055*_4775 spacer, Spe^R	This study

pTH14357	pSGKP with *rcsB* spacer, Spe^R	This study
pTH14366	pSGKP with *VK055*_4961 spacer, Spe^R	This study
pTH14362	pSGKP with *VK055*_4995 spacer, Spe^R	This study
pTH14361	pSGKP with *VK055*_5004 spacer, Spe^R	This study
pTH14396	pSGKP with Δ*ompR* spacer, Spe^R	This study
pTH14363	pSGKP with *wza* spacer, Spe^R	This study
pTH14807	pSGKP with *envZ* spacer, Spe^R	This study
pTH15056	pSGKP with *VK055*_4983 spacer, Spe^R	This study
pTH15304	pSGKP with *VK055*_4481 spacer, Spe^R	This study
pTH15094	pSGKP with *VK055*_3555 spacer, Spe^R	This study
pTH16138	pSGKP with *VK055*_1149 spacer, Spe^R	This study
pTH15451	pSGKP with *F-type operon* spacer, Spe^R	This study
pTH16265	pSGKP with *gcvTHP* spacer, Spe^R	This study

Spe^R: spectinomycin resistance; Apr^R: apramycin resistance; Kan^R: kanamycin resistance; ATCC: American Type Culture Collection

1. Wang Y, Wang S, Chen W, Song L, Zhang Y, Shen Z, Yu F, Li M, Ji Q. 2018. CRISPR-Cas9 and CRISPR-assisted cytidine deaminase enable precise and efficient genome editing in *Klebsiella pneumoniae*. Appl Environ Microbiol 84:e01834-18.

Table S4. Primers used in this study

ID	Sequences (5'-3')	Descriptions
Pr16207	CGTCTGCGAACGCTGACTGAACTAAGC	Forward primer to amplify the upstream of *VK055*_0032
Pr16208	ACACAGGCCATGGCGAATATCTTCCAGAA TGCGTCGTATCCCTG	Reverse primer to amplify the upstream of *VK055*_0032
Pr16209	CGACGCATTCTGGAAGATATTCGCCATGG CCTGTGTAATGCGGA	Forward primer to amplify the downstream of *VK055*_0032
Pr16210	CGGCTTAGTCTGCACATTGATTCGACG	Reverse primer to amplify the downstream of *VK055*_0032
Pr16428	TAGTATTCACCACTTCCTGCGGCG	Forward sequence to form spacer targeting *VK055*_0032
Pr16429	AAACCGCCGCAGGAAGTGGTGAAT	Reverse sequence to form spacer targeting *VK055*_0032
Pr16505	TCTGGTGGGCAACATCGGGCTT	Forward primer to amplify the upstream of *VK055*_0233
Pr16506	GACGACCATCCGATGCTGAAGAAAATGAA GCTCAAATCCCGCGT	Reverse primer to amplify the upstream of *VK055*_0233
Pr16507	TTTGAGCTTCATTTTCTTCAGCATCGGATG GTCGTCAATGAG	Forward primer to amplify the downstream of *VK055*_0233
Pr16508	CGCGATATCGAGGTACGGGTTTAC	Reverse primer to amplify the downstream of *VK055*_0233
Pr16470	TAGTGCTTAATACCATCTCTCCGG	Forward sequence to form spacer targeting *VK055*_0233
Pr16471	AAACCCGGAGAGATGGTATTAAGC	Reverse sequence to form spacer targeting *VK055*_0233
Pr16402	TCCAGCGGGCCGGTATTGAGAT	Forward primer to amplify the upstream of *VK055*_0249
Pr16403	TTGGCTAATTTGGTTAAGGCGGAAAACGG GTTCGTCTTCAACTAT	Reverse primer to amplify the upstream of *VK055*_0249
Pr16404	GACGAACCCGTTTTCCGCCTTAACCAAAT TAGCCAACGCTGCAC	Forward primer to amplify the downstream of *VK055*_0249
Pr16405	CCAGATATCGGCGCTGAGGAC	Reverse primer to amplify the downstream of *VK055*_0249
Pr16482	TAGTCCAGTTGTCGATAATGAACG	Forward sequence to form spacer targeting *VK055*_0249
Pr16483	AAACCGTTCATTATCGACAACTGG	Reverse sequence to form spacer targeting *VK055*_0249
Pr16212	GGTGATCCTGCTGCACGGCTG	Forward primer to amplify the upstream of *VK055*_0682
Pr16213	CGGTACCGACAGCACATACAGATAGTCGA CAATCAGTTCCCGGAT	Reverse primer to amplify the upstream of *VK055*_0682
Pr16214	CTGATTGTCGACTATCTGTATGTGCTGTCG GTACCGGTCTCCAT	Forward primer to amplify the downstream of *VK055*_0682
Pr16215	GGCCGACATCAGCATAATCGCAG	Reverse primer to amplify the downstream of *VK055*_0682
Pr16446	TAGTGCCCAACCTGCAGATCACCG	Forward sequence to form spacer targeting *VK055*_0682
Pr16447	AAACCGGTGATCTGCAGGTTGGGC	Reverse sequence to form spacer targeting *VK055*_0682
Pr16217	GGTAAATCTCATCAACCCCGCGG	Forward primer to amplify the upstream of *VK055*_0953
Pr16218	TTCGGCGACCAGACGGTAAATCATCTCCT GGGCCTGACTGAGC	Reverse primer to amplify the upstream of *VK055*_0953
Pr16219	CAGGCCCAGGAGATGATTTACCGTCTGGT CGCCGAACAGTAC	Forward primer to amplify the downstream of *VK055*_0953
Pr16220	AGGAGAGCCCGATGTCCGATAAC	Reverse primer to amplify the downstream of *VK055*_0953
Pr16448	TAGTCGTTATCATGATTACCTCGG	Forward sequence to form spacer targeting *VK055*_0953
Pr16449	AAACCCGAGGTAATCATGATAACG	Reverse sequence to form spacer targeting *VK055*_0953
Pr16222	CGGTCAAATCACACACCAACGGTC	Forward primer to amplify the upstream of *VK055*_0985
Pr16223	TTTGTTGCGAACGGTCTTGCCTACCTCAGG GTCATCCTCAAC	Reverse primer to amplify the upstream of *VK055*_0985
Pr16224	GATGACCCTGAGGTAGGCAAGACCGTTCG CAACAAAGGCTACC	Forward primer to amplify the downstream of *VK055*_0985

Pr16225	TCAAATCGTCAAGCGACTGCCGT	Reverse primer to amplify the downstream of *VK055*_0985
Pr16450	TAGTCCACAGCAGATCGAAATCGG	Forward sequence to form spacer targeting *VK055*_0985
Pr16451	AAACCCGATTTCGATCTGCTGTGG	Reverse sequence to form spacer targeting *VK055*_0985
Pr16232	GAGCCGGCGCATCATGACGTAAC	Forward primer to amplify the upstream of *VK055*_1326
Pr16233	GACGTCCTGTGGGTATTCCTGCAGCTGAA CTTTGAGGTGGTGAC	Reverse primer to amplify the upstream of *VK055*_1326
Pr16234	CTCAAAGTTCAGCTGCAGGAATACCCACA GGACGTCATCACCAC	Forward primer to amplify the downstream of *VK055*_1326
Pr16235	TGCGGCTTATCTGCTCCAGCATC	Reverse primer to amplify the downstream of *VK055*_1326
Pr16454	TAGTGAAACCCTGATCCGTAACCG	Forward sequence to form spacer targeting *VK055*_1326
Pr16455	AAACCGGTTACGGATCAGGGTTTC	Reverse sequence to form spacer targeting *VK055*_1326
Pr16237	CTTCAGGGTCAGCGTTTCCAGATAC	Forward primer to amplify the upstream of *VK055*_1598
Pr16238	GAGGATATAGCCACGCTGGTCTGGGGAAA AGGCTATAAGTTCGC	Reverse primer to amplify the upstream of *VK055*_1598
Pr16239	ATAGCCTTTTCCCCAGACCAGCGTGGCTA TATCCTCGTCATCTTC	Forward primer to amplify the downstream of *VK055*_1598
Pr16240	GTTTTTGCTGCCCTGAAGCCCTTTATC	Reverse primer to amplify the downstream of *VK055*_1598
Pr16456	TAGTTACAGCAGGTCGAATTCACG	Forward sequence to form spacer targeting *VK055*_1598
Pr16457	AAACCGTGAATTCGACCTGCTGTA	Reverse sequence to form spacer targeting *VK055*_1598
Pr16372	GACTTCGCGAGCGGATAACTTCTCAC	Forward primer to amplify the upstream of *VK055*_1724
Pr16373	GCATATAGCCAAAGCCCCACTTTGCATCG CCAGAATTAACCCCT	Reverse primer to amplify the upstream of *VK055*_1724
Pr16374	ATTCTGGCGATGCAAAGTGGGGCTTTGGC TATATGCTGGCTAAC	Forward primer to amplify the downstream of *VK055*_1724
Pr16375	TCCATGCGCACGAAGGCTTTACTG	Reverse primer to amplify the downstream of *VK055*_1724
Pr16480	TAGTAGCCTGATCGTACTCGACCT	Forward sequence to form spacer targeting *VK055*_1724
Pr16481	AAACAGGTCGAGTACGATCAGGCT	Reverse sequence to form spacer targeting *VK055*_1724
Pr16242	GGACTACTGGTGGTGGAACCGGAG	Forward primer to amplify the upstream of *VK055*_1819
Pr16243	TCGCAGATGACCCATATACAGAAAGCGTC GGATAGCGTGTTCG	Reverse primer to amplify the upstream of *VK055*_1819
Pr16244	GCTATCCGACGCTTTCTGTATATGGGTCAT CTGCGACAAAAGCTGG	Forward primer to amplify the downstream of *VK055*_1819
Pr16245	CAGGAGCATTACAACGAACTGGCG	Reverse primer to amplify the downstream of *VK055*_1819
Pr16458	TAGTGAAACTCGATAGGCGTCAGG	Forward sequence to form spacer targeting *VK055*_1819
Pr16459	AAACCCTGACGCCTATCGAGTTTC	Reverse sequence to form spacer targeting *VK055*_1819
Pr16542	CTCATCGACGGTGAAGATCAGCGT	Forward primer to amplify the upstream of *VK055*_2216
Pr16543	GCTCCAATCCGCGAAATGCATGATCGTAT GGTGCAAACGGTTCG	Reverse primer to amplify the upstream of *VK055*_2216
Pr16544	TTGCACCATACGATCATGCATTTCGCGGA TTGGAGCTTCATCTTC	Forward primer to amplify the downstream of *VK055*_2216
Pr16545	CAGATGCACCGATTTCGCTTTGCC	Reverse primer to amplify the downstream of *VK055*_2216
Pr16472	TAGTTCCGTCGGTCCCATATCCAG	Forward sequence to form spacer targeting *VK055*_2216
Pr16473	AAACCTGGATATGGGACCGACGGA	Reverse sequence to form spacer targeting *VK055*_2216
Pr16387	CAGTCGATCGACAGCGTCATTTCG	Forward primer to amplify the upstream of *VK055*_2521
Pr16388	AATTTTTCCGTACAGCATCAGTTCGCTCTC ATCTTCAATGATTAGCAC	Reverse primer to amplify the upstream of *VK055*_2521
Pr16389	GAAGATGAGAGCGAACTGATGCTGTACGG AAAAATTGGCCATCC	Forward primer to amplify the downstream of *VK055*_2521

Pr16390	GCTATGTCCCACAATTGACCGAGGC	Reverse primer to amplify the downstream of *VK055*_2521
Pr16418	TAGTCGTCACGCTGATGACCGACC	Forward sequence to form spacer targeting *VK055*_2521
Pr16419	AAACGGTCGGTCATCAGCGTGACG	Reverse sequence to form spacer targeting *VK055*_2521
Pr18260	CTTCTACGATGCGCCACAGA	Forward primer to amplify the upstream of *VK055*_2547
Pr18261	TCTGCTCAGGATAGGTCTGCTCGCCAGCATAGGTTCGTCTT	Reverse primer to amplify the upstream of *VK055*_2547
Pr18262	AAGACGAACCTATGCTGGCGAGCAGACCTATCCTGAGCAGA	Forward primer to amplify the downstream of *VK055*_2547
Pr18263	AAGAGTGCATTGGCTGGTGG	Reverse primer to amplify the downstream of *VK055*_2547
Pr18240	TAGTATACCAATGACGTCGAACTG	Forward sequence to form spacer targeting the *VK055*_2547
Pr18241	AAACCAGTTCGACGTCATTGGTAT	Reverse sequence to form spacer targeting the *VK055*_2547
Pr16354	CACGACCAAATACCAGGGCAGTATG	Forward primer to amplify the upstream of *VK055*_2575
Pr16355	GATGATTTCCGGGGTATCGTTGCGTGTTACCAACTCGTCTTCAAC	Reverse primer to amplify the upstream of *VK055*_2575
Pr16356	GAGTTGGTAACACGCAACGATACCCCGGAAATCATCGCTACCATTC	Forward primer to amplify the downstream of *VK055*_2575
Pr16357	GAGGTCAACGGTAACGTATTAAGCGTGG	Reverse primer to amplify the downstream of *VK055*_2575
Pr16476	TAGTTTCGCCGTTCGGGCTAACCA	Forward sequence to form spacer targeting *VK055*_2575
Pr16477	AAACTGGTTAGCCCGAACGGCGAA	Reverse sequence to form spacer targeting *VK055*_2575
Pr16314	CGAGGATATTATCAGTAAACCGCGCC	Forward primer to amplify the upstream of *VK055*_2578
Pr16315	GCTGATACCCTGGTTTACCCTGAAAAGGCGCCAATCAATACCCATC	Reverse primer to amplify the upstream of *VK055*_2578
Pr16316	GATTGGCGCCTTTTCAGGGTAAACCAGGGTATCAGCGATACTCTGTTC	Forward primer to amplify the downstream of *VK055*_2578
Pr16317	GACAGCAGATTTCGACAAGCGGC	Reverse primer to amplify the downstream of *VK055*_2578
Pr16460	TAGTACGTCGAGGATGGCGACATC	Forward sequence to form spacer targeting *VK055*_2578
Pr16461	AAACGATGTCGCCATCCTCGACGT	Reverse sequence to form spacer targeting *VK055*_2578
Pr16377	GGCTTAAGGCGATTTCGGTTTGCG	Forward primer to amplify the upstream of *VK055*_2704
Pr16378	GAGTACCTGACCAAAGGGCGGCTGCGCGCCAAAATTGATAACGAC	Reverse primer to amplify the upstream of *VK055*_2704
Pr16379	AATTTTGGCGCGCAGCCGCCCTTTGGTCAGGTACTCTCCGGTTTTC	Forward primer to amplify the downstream of *VK055*_2704
Pr16380	GCAGCAGCTGCAGGGCGTTATTG	Reverse primer to amplify the downstream of *VK055*_2704
Pr16462	TAGTCGTTGGGCACCATTGAGCAT	Forward sequence to form spacer targeting *VK055*_2704
Pr16463	AAACATGCTCAATGGTGCCCAACG	Reverse sequence to form spacer targeting *VK055*_2704
Pr16319	CACCCAGGCGAGGTCGATATCCAC	Forward primer to amplify the upstream of *VK055*_2984
Pr16320	ATAGCGATGGGCGAACTGATTAAAACCCTGCGCGGTCTGGGCTATG	Reverse primer to amplify the upstream of *VK055*_2984
Pr16321	ACCGCGCAGGGTTTTAATCAGTTCGCCCATCGCTATGTCGTCAT	Forward primer to amplify the downstream of *VK055*_2984
Pr16322	GAACGGCTGGCGACGAAGGATTTATC	Reverse primer to amplify the downstream of *VK055*_2984
Pr16426	TAGTAAAGCGGTACTGCGACGCAG	Forward sequence to form spacer targeting *VK055*_2984
Pr16427	AAACCTGCGTCGCAGTACCGCTTT	Reverse sequence to form spacer targeting *VK055*_2984
Pr16324	ATTTTCTCGCCCGTCATCATATTCCG	Forward primer to amplify the upstream of *VK055*_3087
Pr16325	ATCAGCCACTGCACGATCGAAAAAACGGGCGGCAACAAAACTGAAG	Reverse primer to amplify the upstream of *VK055*_3087
Pr16326	GTTGCCGCCCGTTTTTTCGATCGTGCAGTGGCTGATGTCATCATC	Forward primer to amplify the downstream of *VK055*_3087

Pr16327	CTGGAGATCTATCGCCATTTTCGCC	Reverse primer to amplify the downstream of *VK055*_3087
Pr16424	TAGTGCTGTGCGATATCCGTATGG	Forward sequence to form spacer targeting *VK055*_3087
Pr16425	AAACCCATACGGATATCGCACAGC	Reverse sequence to form spacer targeting *VK055*_3087
Pr16382	AATGTCCACCAGCGAATCCACCAG	Forward primer to amplify the upstream of *VK055*_3275
Pr16383	CGGATGGCCATCTTTGCGATCGAGCAGCT CTTTTAACAGGGAGGTG	Reverse primer to amplify the upstream of *VK055*_3275
Pr16384	TTAAAAGAGCTGCTCGATCGCAAAGATGG CCATCCGTGGTTTAAAAC	Forward primer to amplify the downstream of *VK055*_3275
Pr16385	GCATTGTCCAACACTTCGTTCCATAGC	Reverse primer to amplify the downstream of *VK055*_3275
Pr16464	TAGTGTTATCATGCTAACAGCGCG	Forward sequence to form spacer targeting *VK055*_3275
Pr16465	AAACCGCGCTGTTAGCATGATAAC	Reverse sequence to form spacer targeting *VK055*_3275
Pr16510	ATGATGACCTGGCGGTGCACTTTG	Forward primer to amplify the upstream of *VK055*_3297
Pr16511	TTTGTGTCCCTGGGTATGTTCAAGCACCCA GCGGATGGAGCTATC	Reverse primer to amplify the upstream of *VK055*_3297
Pr16512	ATCCGCTGGGTGCTTGAACATACCCAGGG ACACAAACAGGAGGCTG	Forward primer to amplify the downstream of *VK055*_3297
Pr16513	GAGCTCAATCCTGACCGCCTGAG	Reverse primer to amplify the downstream of *VK055*_3297
Pr16436	TAGTCTGCTGATAAGCGCTGACCG	Forward sequence to form spacer targeting *VK055*_3297
Pr16437	AAACCGGTCAGCGCTTATCAGCAG	Reverse sequence to form spacer targeting *VK055*_3297
Pr16527	GCGATCATCACTACCGACGTTGGCC	Forward primer to amplify the upstream of *VK055*_3393
Pr16528	CAGCTTTTCCAGCAGGTTAAAGCCGGAGC GGACAATCAAATGATCG	Reverse primer to amplify the upstream of *VK055*_3393
Pr16529	ATTGTCCGCTCCGGCTTTAACCTGCTGGAA AAGCTGGGCGTCAG	Forward primer to amplify the downstream of *VK055*_3393
Pr16530	GACAGCTGTTCTATCAGTTGACCGCTC	Reverse primer to amplify the downstream of *VK055*_3393
Pr16466	TAGTTATCGCCGCGGTACGCACGG	Forward sequence to form spacer targeting *VK055*_3393
Pr16467	AAACCCGTGCGTACCGCGGCGATA	Reverse sequence to form spacer targeting *VK055*_3393
Pr16520	GCAATAAACGGCACGGTGTTCCC	Forward primer to amplify the upstream of *ompR*
Pr16521	GTAGCCCAGACCCCAGACCTGGAAGCCCT GCTCGGTCAGATAAC	Reverse primer to amplify the upstream of *ompR*
Pr16522	ACCGAGCAGGGCTTCCAGGTCTGGGGTCT GGGCTACGTCTTCG	Forward primer to amplify the downstream of *ompR*
Pr16523	GATTTCCCGCTCATAGCCGCTCTC	Reverse primer to amplify the downstream of *ompR*
Pr16478	TAGTAAGCTGATGAACCTCGCCCG	Forward sequence to form spacer targeting *ompR*
Pr16479	AAACCGGGCGAGGTTCATCAGCTT	Reverse sequence to form spacer targeting *ompR*
Pr16392	GAAAATAGGGGTTCACCAGCAGGC	Forward primer to amplify the upstream of *VK055*_3990
Pr16393	ATTCGCAACCTTCTCGACATGGATAAATT AGGCTGCAGCACCCTG	Reverse primer to amplify the upstream of *VK055*_3990
Pr16394	GCAGCCTAATTTATCCATGTCGAGAAGGT TGCGAATGGCGATAC	Forward primer to amplify the downstream of *VK055*_3990
Pr16395	GAAAACCAACTTTGTGGCCACTATCGG	Reverse primer to amplify the downstream of *VK055*_3990
Pr16444	TAGTGTCGATATTCCGGAGCTCAG	Forward sequence to form spacer targeting *VK055*_3990
Pr16445	AAACCTGAGCTCCGGAATATCGAC	Reverse sequence to form spacer targeting *VK055*_3990
Pr16329	TGCTGCACATCATCCAGTTGGGCG	Forward primer to amplify the upstream of *VK055*_4034
Pr16330	CTTATCGGCGACGGCATCGCAAGCTCGGC AGCGACTTTATCCGTAC	Reverse primer to amplify the upstream of *VK055*_4034
Pr16331	AGTCGCTGCCGAGCTTGCGATGCCGTCGC CGATAAGCTTGTCATC	Forward primer to amplify the downstream of *VK055*_4034

Pr16332	GTTGACGGAATTGGGTCGGGCTTTG	Reverse primer to amplify the downstream of *VK055*_4034
Pr16422	TAGTGGATCAGCACCGGCTCATGG	Forward sequence to form spacer targeting *VK055*_4034
Pr16423	AAACCCATGAGCCGGTGCTGATCC	Reverse sequence to form spacer targeting *VK055*_4034
Pr18264	AAAGGGGGACTGCTACTCAAA	Forward primer to amplify the upstream of *VK055*_4175
Pr18265	TCCGATGAGTTCTACAAGCGTAGGACCACGCTGATATTTCTCT	Reverse primer to amplify the upstream of *VK055*_4175
Pr18266	AGAGAAATATCAGCGTGGTCCTACGCTTGTAGAACTCATCGGA	Forward primer to amplify the downstream of *VK055*_4175
Pr18267	ATTGTTGGCGGGATGTTCCT	Reverse primer to amplify the downstream of *VK055*_4175
Pr18246	TAGTCTGCCAGATATCGACGGGAT	Forward sequence to form spacer targeting the *VK055*_4175
Pr18247	AAACATCCCGTCGATATCTGGCAG	Reverse sequence to form spacer targeting the *VK055*_4175
Pr16495	GGGGCATAATAAGGATTGACCAGAG	Forward primer to amplify the upstream of *VK055*_4178
Pr16496	ATTATTGACGATCATCCATATAAAAGAAGGCTTATGGGGAAACTCAAC	Reverse primer to amplify the upstream of *VK055*_4178
Pr16497	CATAAGCCTTCTTTTATATGGATGATCGTCAATAATGACTGCACTC	Forward primer to amplify the downstream of *VK055*_4178
Pr16498	CTTCCCTGCCAGCCCATATTAAG	Reverse primer to amplify the downstream of *VK055*_4178
Pr16474	TAGTTGACAATGCTACCAGCGTAG	Forward sequence to form spacer targeting *VK055*_4178
Pr16475	AAACCTACGCTGGTAGCATTGTCA	Reverse sequence to form spacer targeting *VK055*_4178
Pr18268	CCCGCAACCAGTGAAGAGATA	Forward primer to amplify the upstream of *VK055*_4202
Pr18269	ACCGGCACATCGCATTATCCAATCGCGCTTAAATTTGCCGT	Reverse primer to amplify the upstream of *VK055*_4202
Pr18270	ACGGCAAATTTAAGCGCGATTGGATAATGCGATGTGCCGGT	Forward primer to amplify the downstream of *VK055*_4202
Pr18271	CAGGGAAGATATGCGCGAGA	Reverse primer to amplify the downstream of *VK055*_4202
Pr18248	TAGTCGAAGCGTTAATGGCTGTTG	Forward sequence to form spacer targeting the *VK055*_4202
Pr18249	AAACCAACAGCCATTAACGCTTCG	Reverse sequence to form spacer targeting the *VK055*_4202
Pr16537	GTATGCGTGAAGGTACCGAGCTGTTG	Forward primer to amplify the upstream of *VK055*_4622
Pr16538	GTTGCCCTTGGTAATTTGATCGTCATCCACCAGCAGTAAACGCG	Reverse primer to amplify the upstream of *VK055*_4622
Pr16539	CTGCTGGTGGATGACGATCAAATTACCAAGGGCAACGTCACTCACG	Forward primer to amplify the downstream of *VK055*_4622
Pr16540	GGCGTGCTGGCGAACGTCTTTATC	Reverse primer to amplify the downstream of *VK055*_4622
Pr16442	TAGTGGTCTTCACGAAACTCGCCG	Forward sequence to form spacer targeting *VK055*_4622
Pr16443	AAACCGGCGAGTTTCGTGAAGACC	Reverse sequence to form spacer targeting*VK055*_4622
Pr16500	GCTTTAGTAGGAGATGTCGGGGGAC	Forward primer to amplify the upstream of *VK055*_4775
Pr16501	TGGCTTATTAACACCCATTTTCAGGTTCCGGTCAGTCGCAGTAAAG	Reverse primer to amplify the upstream of *VK055*_4775
Pr16502	ACTGACCGGAACCTGAAAATGGGTGTTAATAAGCCAGGAGAGCTCC	Forward primer to amplify the downstream of *VK055*_4775
Pr16503	TGGCGATCACCAATACCGAGCAC	Reverse primer to amplify the downstream of *VK055*_4775
Pr16468	TAGTGCATGACGAATGACTCCCGG	Forward sequence to form spacer targeting *VK055*_4775
Pr16469	AAACCCGGGAGTCATTCGTCATGC	Reverse sequence to form spacer targeting *VK055*_4775
Pr16547	GATATTGCTGTCGAAACCGAGCCG	Forward primer to amplify the upstream of *rcsB*
Pr16548	CATCCGATCGTACTGTTCAACTACCTCTCTTCCGTCTCGCTGAGC	Reverse primer to amplify the upstream of *rcsB*
Pr16549	GACGGAAGAGAGGTAGTTGAACAGTACGATCGGATGGTCATCGG	Forward primer to amplify the downstream of *rcsB*

Pr16550	CCCTCGACGTCTGTCAGGATGAG	Reverse primer to amplify the downstream of *rcsB*
Pr16420	TAGTCAGGCGAGAGACGCTTTCCG	Forward sequence to form spacer targeting *rcsB*
Pr16421	AAACCGGAAAGCGTCTCTCGCCTG	Reverse sequence to form spacer targeting *rcsB*
Pr16334	TATCTCCTCCTCGTATACGCAACGGGC	Forward primer to amplify the upstream of *VK055*_4961
Pr16335	CTCCGCCTGACCATTCTCGATATCGCGCTG TGTCTCCAGCAAAATGC	Reverse primer to amplify the upstream of *VK055*_4961
Pr16336	GAGACACAGCGCGATATCGAGAATGGTCA GGCGGAGCTGCTGATGC	Forward primer to amplify the downstream of *VK055*_4961
Pr16337	AACGCAATGTCTTCTCCGGCATCC	Reverse primer to amplify the downstream of *VK055*_4961
Pr16440	TAGTGCAGCAGCCAGATACGACTG	Forward sequence to form spacer targeting *VK055*_4961
Pr16441	AAACCAGTCGTATCTGGCTGCTGC	Reverse sequence to form spacer targeting *VK055*_4961
Pr16339	TCGGCTCATATTTGTGGACGATGTTGCC	Forward primer to amplify the upstream of *VK055*_4995
Pr16340	CCACGCATCCTTATCGTGTATGGCGTTGGC TACCGCTGGGAAGC	Reverse primer to amplify the upstream of *VK055*_4995
Pr16341	GCGGTAGCCAACGCCATACACGATAAGGA TGCGTGGGGTGTTTTCATC	Forward primer to amplify the downstream of *VK055*_4995
Pr16342	CAAAGTGATCGCCTCGCCGGTAGAG	Reverse primer to amplify the downstream of *VK055*_4995
Pr16432	TAGTAGCCCGCTGATTGTCGACGA	Forward sequence to form spacer targeting *VK055*_4995
Pr16433	AAACTCGTCGACAATCAGCGGGCT	Reverse sequence to form spacer targeting *VK055*_4995
Pr16344	AGCCCTTCGAGAATACCGTGCACTAC	Forward primer to amplify the upstream of *VK055*_5004
Pr16345	GATAACGCCGATCTGCTGCTGCCCGTTCG CGCAATCTTTGGTAAG	Reverse primer to amplify the upstream of *VK055*_5004
Pr16346	GATTGCGCGAACGGGCAGCAGCAGATCG GCGTTATCTTCAATTATCGC	Forward primer to amplify the downstream of *VK055*_5004
Pr16347	TGGCTGTGGCGCAAAAAAGCTATCG	Reverse primer to amplify the downstream of *VK055*_5004
Pr16430	TAGTCCAGCAGGAATATCGCCTGG	Forward sequence to form spacer targeting *VK055*_5004
Pr16431	AAACCCAGGCGATATTCCTGCTGG	Reverse sequence to form spacer targeting *VK055*_5004
Pr16414	TAGTCCAGACCTGGAAGCCCTGCT	Forward sequence to form spacer targeting Δ*ompR*
Pr16415	AAACAGCAGGGCTTCCAGGTCTGG	Reverse sequence to form spacer targeting Δ*ompR*
Pr16516	GGCAGCATCAGAGCCAGCACCATCAGGTG GAAGGATTCACG	Reverse primer to amplify the upstream of *ompR* site mutation D55A
Pr16517	GATGGTGCTGGCTCTGATGCTGCCGGGCG AAGATGGTCTCTC	Forward primer to amplify the downstream of *ompR* site mutation D55A
Pr16515	ATGATCGCCAGGGTATAGCGGAACAG	Reverse primer to amplify the downstream of *ompR* site mutation D55A
Pr16434	TAGTCAGCAATTGTCATACCACTG	Forward sequence to form spacer targeting *wza*
Pr16435	AAACCAGTGGTATGACAATTGCTG	Reverse sequence to form spacer targeting *wza*
Pr16532	GTCCATGGCAGTACCAAATCTCCG	Forward primer to amplify the upstream of *wza*b
Pr16533	ACGTAGACTGTTTAATCCCTGACCAGG	Reverse primer to amplify the upstream of *wza*b
Pr16534	CCTGATCCTTATAGGAAAAGTGAAGAGGC C	Forward primer to amplify the downstream of *wza*b
Pr16535	AGCCTCTAACGACAAATCGACGG	Reverse primer to amplify the downstream of *wza*b
Pr17076	TAGTCGTATTATCGACAATCACAA	Forward sequence to form spacer targeting *envZ*
Pr17077	AAACTTGTGATTGTCGATAATACG	Reverse sequence to form spacer targeting *envZ*
Pr17080	GGCGCCACAGGTAGTCCATC	Forward primer to amplify the upstream of *envZ*

Pr17081	CGGCGCCAGCATCCGTGACGATCAGGAGCAGTGTG	Reverse primer to amplify the upstream of *envZ*
Pr17082	CTGCTCCTGATCGTCACGGATGCTGGCGCCGATGAAAC	Forward primer to amplify the downstream of *envZ*
Pr17083	AGCAGTACATCTTGGCTAAGGAGC	Reverse primer to amplify the downstream of *envZ*
Pr17242	GCTGTCTACCACGTCGCCTTG	Forward primer to amplify the upstream of *VK055*_4983
Pr17243	TCCAGCAGTTCAGGACCGATATGAAGCCATGTGAATTG	Reverse primer to amplify the upstream of *VK055*_4983
Pr17244	TTCATATCGGTCCTGAACTGCTGGAAGCGCTTAG	Forward primer to amplify the downstream of *VK055*_4983
Pr17245	GCTGTACCAGTGATAGCCATCAATC	Reverse primer to amplify the downstream of *VK055*_4983
Pr17246	TAGTATGCGGCAAACCGCACCCGG	Forward sequence to form spacer targeting *VK055*_4983
Pr17247	AAACCCGGGTGCGGTTTGCCGCAT	Reverse sequence to form spacer targeting *VK055*_4983
Pr17417	TAGTGAAGCGCTATCTAAGCCGGG	Forward sequence to form spacer targeting *VK055*_4481
Pr17418	AAACCCCGGCTTAGATAGCGCTTC	Reverse sequence to form spacer targeting *VK055*_4481
Pr17419	TAAATACAGCGTTTCGCAGCTG	Forward primer to amplify the upstream of *VK055*_4481
Pr17420	CTCAACGTTGTGACGATATCATCCCGCTCAGCGGAG	Reverse primer to amplify the upstream of *VK055*_4481
Pr17421	TGAGCGGGATGATATCGTCACAACGTTGAGCTTATCG	Forward primer to amplify the downstream of *VK055*_4481
Pr17422	GCGCATGATGGAGCCTTTACC	Reverse primer to amplify the downstream of *VK055*_4481
Pr17254	TAGTGCTCCGCATAGATAGCGCCG	Forward sequence to form spacer targeting *VK055*_3555
Pr17255	AAACCGGCGCTATCTATGCGGAGC	Reverse sequence to form spacer targeting *VK055*_3555
Pr17256	GGAACCGATCCCATTGTGGAG	Forward primer to amplify the upstream of *VK055*_3555
Pr17257	CCAAGTGGAAGGTAAACACGATAGGCATGATGTACCAC	Reverse primer to amplify the upstream of *VK055*_3555
Pr17258	CTATCGTGTTTACCTTCCACTTGGCGCTCATC	Forward primer to amplify the downstream of *VK055*_3555
Pr17259	GGTTCGTGTTCGAACACATCGAG	Reverse primer to amplify the downstream of *VK055*_3555
Pr18365	ACAGTCCACCAGCACAATCT	Forward primer to amplify the upstream of *pspABCD*
Pr18366	CTCGTTTTGCCGACATCGTGATGAAATAATGCCGCCGTGG	Reverse primer to amplify the upstream of *pspABCD*
Pr18367	CCACGGCGGCATTATTTCATCACGATGTCGGCAAAACGAG	Forward primer to amplify the downstream of *pspABCD*
Pr18368	TTCGCTAAAGCCGGGAAACA	Reverse primer to amplify the downstream of *pspABCD*
Pr18369	TAGTTGGCTGCACTACAACAATCG	Forward sequence to form spacer targeting *pspABCD*
Pr18370	AAACCGATTGTTGTAGTGCAGCCA	Reverse sequence to form spacer targeting *pspABCD*
Pr17827	TAGTCGACACGCGACATACCTCAA	Forward sequence to form spacer targeting *F-type atp operon*
Pr17828	AAACTTGAGGTATGTCGCGTGTCG	Reverse sequence to form spacer targeting *F-type atp operon*
Pr18282	GAATGAGATGCTGGTGCGTC	The forward primer to amplify the upstream of *F-type atp operon*
Pr18283	TCACTTCATCCGCTCGTCAGTTTCAGCGTTCTGTGAGCGG	The reverse primer to amplify the upstream of *F-type atp operon*
Pr18284	CCGCTCACAGAACGCTGAAACTGACGAGCGGATGAAGTGA	The forward primer to amplify the downstream of *F-type atp operon*
Pr18285	ACGTCGCCATAGAGCATCAG	The reverse primer to amplify the downstream of *F-type atp operon*
Pr18496	TAGTACGTTCTGCATTCCGCACGG	Forward sequence to form spacer targeting *gcvTHP*

Pr18497	AAACCCGTGCGGAATGCAGAACGT	Reverse sequence to form spacer targeting *gcvTHP*
Pr18490	GTTTTATGGATGCCGCCGAG	The forward primer to amplify the upstream of *gcvTHP*
Pr18491	GGTCGCCGTAGACATCATCCCGGAGTCTGTTGAGCCATCT	The reverse primer to amplify the upstream of *gcvTHP*
Pr18492	AGATGGCTCAACAGACTCCGGGATGATGTCTACGGCGACC	The forward primer to amplify the downstream of *gcvTHP*
Pr18493	CGGAGTCTGTTGAGCCATCT	The reverse primer to amplify the downstream of *gcvTHP*
Pr18329	ACCGTGTGCTTCTCAAATGCCTAGCTATACTGATTTCGTCAGACTCAC	Forward primer to amplify the *rspL* promoter for pTH16235 construction
Pr18330	CACTAGTGATGGTGATGGTGATGATTATGAGGACGCAGAATTTTAGG	Reverse primer to amplify the *rspL* promoter for pTH16235 construction
Pr18331	CTGCGTCCTCATAATCATCACCATCACCATCACTAGTGAGGCCAGTTTGCTCAGG	Forward primer to amplify the backbone from pTH15652 for pTH16235 construction
Pr18332	GAAATCAGTATAGCTAGGCATTTGAGAAGCACACGG	Reverse primer to amplify the backbone from pTH15652 for pTH16235 construction
Pr18341	CCTAAAATTCTGCGTCCTCATAATCAATTCACCATTGTCGGTATTTTTGG	Forward primer to amplify *fyuA*
Pr18342	CACTAGTGATGGTGATGGTGATGGAAGAAATCAATTCGCGTATTGATAC	Reverse primer to amplify *fyuA*
Pr18343	ACGCGAATTGATTTCTTCCATCACCATCACCATCACTAGTG	Forward primer to amplify the backbone from pTH16235
Pr18344	CCAAAAATACCGACAATGGTGAATTGATTATGAGGACGCAGAATTTTAGG	Reverse primer to amplify the backbone from pTH16235
Pr18349	CCTAAAATTCTGCGTCCTCATAATCATGGCGCAAAATCGCCGAC	Forward primer to amplify *gcvTHP*
Pr18350	CACTAGTGATGGTGATGGTGATGCTGGTATTCGCTCATCGGCAC	Reverse primer to amplify *gcvTHP*
Pr18351	GTGCCGATGAGCGAATACCAGCATCACCATCACCATCACTAGTG	Forward primer to amplify the backbone from pTH16235
Pr18352	GTCGGCGATTTTGCGCCATGATTATGAGGACGCAGAATTTTAGG	Reverse primer to amplify the backbone from pTH16235
Pr18353	CTAAAATTCTGCGTCCTCATAATGCCGGTAATCAAGAAAAAGAAGGC	Forward primer to amplify the *VK055*_3379-3380
Pr18354	CACTAGTGATGGTGATGGTGATGATGCAGCTCAGGCCAGTAATC	Reverse primer to amplify the *VK055*_3379-3380
Pr18355	TTACTGGCCTGAGCTGCATCATCACCATCACCATCACTAGTG	Forward primer to amplify the backbone from pTH16235
Pr18356	TCTTTTTCTTGATTACCGGCATTATGAGGACGCAGAATTTTAGG	Reverse primer to amplify the backbone from pTH16235
Pr18357	CCTAAAATTCTGCGTCCTCATAATCAGGCTGGTGGAGAGTATTCC	Forward primer to amplify the *VK055*_0304-0305
Pr18358	TAGTGATGGTGATGGTGATGCTTTGCCGCCAGCTCGCGAC	Reverse primer to amplify the *VK055*_0304-0305
Pr18359	TCGCGAGCTGGCGGCAAAGCATCACCATCACCATCACTAGTG	Forward primer to amplify the backbone from pTH16235
Pr18360	GAATACTCTCCACCAGCCTGATTATGAGGACGCAGAATTTTAGG	Reverse primer to amplify the backbone from pTH16235

Pr18423	CCGTGTGCTTCTCAAATGCCATGCACGCTTTAATTGCCGTG	Forward primer to amplify the *F-type atp operon* with promoter region
Pr18338	CACTAGTGATGGTGATGGTGATGCATCGCTTTTTGGTCAACTCG	Reverse primer to amplify the *F-type atp operon* with promoter region
Pr18426	CACGGCAATTAAAGCGTGCATGGCATTTGAGAAGCACACGG	Forward primer to amplify the backbone from pTH16235
Pr18340	CGAGTTGACCAAAAAAGCGATGCATCACCATCACCATCACTAGTG	Reverse primer to amplify the backbone from pTH16235
Pr17406	CGGTCTGTCAAGTCGGATGTG	Forward primer to amplify the 16S rRNA for qRT-PCR
Pr17407	CGGAAGCCACGCCTCAAG	Reverse primer to amplify the 16S rRNA for qRT-PCR
Pr17950	GTTTGCATCCGACCTTGACG	Forward primer to amplify the *atp*A for qRT-PCR
Pr17951	TCGGCGCATACTGTTTCTGT	Reverse primer to amplify the *atp*A for qRT-PCR
Pr17952	CTGCGTACATTCTCGCTGGT	Forward primer to amplify the *atp*B for qRT-PCR
Pr17953	AACAGCAGACCGAGAACCAC	Reverse primer to amplify the *atp*B for qRT-PCR
Pr17954	CACCGCCATTAAGCCTGGTA	Forward primer to amplify the *atp*C for qRT-PCR
Pr17955	AATCCACATCGCCGTGAGAG	Reverse primer to amplify the *atp*C for qRT-PCR
Pr17956	AGGGCGGTAAAGTTGGTCTG	Forward primer to amplify the *atp*D for qRT-PCR
Pr17957	TACCCTCACGAGTACGCTCA	Reverse primer to amplify the *atp*D for qRT-PCR
Pr17958	GATCGGTATCGGCATCCTCG	Forward primer to amplify the *atp*E for qRT-PCR
Pr17959	ACCCAGACCTACAGCGATCA	Reverse primer to amplify the *atp*E for qRT-PCR
Pr17960	GCGGAAGCCCAGGTAATCAT	Forward primer to amplify the *atp*F for qRT-PCR
Pr17961	TGCAACGATTTTGGTGCGTT	Reverse primer to amplify the *atp*F for qRT-PCR
Pr17962	AAGATCGCAAGCGTCCAGAA	Forward primer to amplify the *atp*G for qRT-PCR
Pr17963	CATGCGCTCCTGCGATTTAC	Reverse primer to amplify the *atp*G for qRT-PCR
Pr17964	CTTTGCCGTCGAACACAACA	Forward primer to amplify the *atp*H for qRT-PCR
Pr17965	CGCCGCAGATAGCGATAAAC	Reverse primer to amplify the *atp*H for qRT-PCR
Pr17966	ATGGCAAGTGGACTGCTGTT	Forward primer to amplify the *atp*I for qRT-PCR
Pr17967	CGGCCTTTTGCTGGTGTATG	Reverse primer to amplify the *atp*I for qRT-PCR
Pr17282	CCAACCTTGCAGGAGTGCTA	Forward primer to amplify the *VK055*_4983 for qRT-PCR
Pr17283	GCCGTCCTGGTACTGATACG	Reverse primer to amplify the *VK055*_4983 for qRT-PCR
Pr18445	CATGAGGTTATCGCCAGCCA	Forward primer to amplify *VK055*_4984 for qRT-PCR
Pr18446	CGCATTTTTCCCGTAGCGTG	Reverse primer to amplify *VK055*_4984 for qRT-PCR
Pr18447	CTGGTTCTGGGCGGTGTATT	Forward primer to amplify *VK055*_4985 for qRT-PCR
Pr18448	TGCTGCGGCGTTTGAATATG	Reverse primer to amplify *VK055*_4985 for qRT-PCR
Pr18449	GGTGCCGCTATTTTCAGCTT	Forward primer to amplify *VK055*_4986 for qRT-PCR
Pr18450	AGCGGCAATAACCTGACGAG	Reverse primer to amplify *VK055*_4986 for qRT-PCR
Pr17284	GCTGGTGGTACATCATGCCT	Forward primer to amplify *VK055*_3555 for qRT-PCR
Pr17285	AGGTCTTCGGTAATGCCTGC	Reverse primer to amplify *VK055*_3555 for qRT-PCR
Pr18451	GCCTCATCCAGTTTACCGCT	Forward primer to amplify *psp*A for qRT-PCR
Pr18452	ATTGGCGAGCTGGAGAACAA	Reverse primer to amplify *psp*A for qRT-PCR
Pr18453	TTTGTCCCTCCAGTTCGGATG	Forward primer to amplify *psp*B for qRT-PCR
Pr18454	CTGACGCAAAATGAGCAGCA	Reverse primer to amplify *psp*B for qRT-PCR
Pr18455	AACTGTGGCGTATCCCGC	Forward primer to amplify *psp*C for qRT-PCR
Pr18456	GAAAATCATCGCCAGCACGG	Reverse primer to amplify *psp*C for qRT-PCR
Pr18457	CAAATGGCAACGCGCCG	Forward primer to amplify *psp*D for qRT-PCR
Pr18458	GATTTAACCGCCCAGCCG	Reverse primer to amplify *psp*D for qRT-PCR
Pr18427	ATGAGCAACGTACCAGCAGAA	Forward primer to amplify *gcv*H for qRT-PCR

Pr18428	GGCAGGTCAACGAAAACCAT	Reverse primer to amplify *gcv*H for qRT-PCR
Pr18429	GAAGAGACCGGCATTAGCGA	Forward primer to amplify *gcv*P for qRT-PCR
Pr18430	ACCTTGCTTTCCGATTCCGT	Reverse primer to amplify *gcv*P for qRT-PCR
Pr18431	GAAATTGCGATGCCGAACGA	Forward primer to amplify *gcv*T for qRT-PCR
Pr18432	ATAGTCCAGCCCATGTTCGC	Reverse primer to amplify *gcv*T for qRT-PCR
Pr18433	CGCCGACAACATTGAAAGCA	Forward primer to amplify *VK055*_3379 for qRT-PCR
Pr18434	CCGACGATGACCTGAATCCC	Reverse primer to amplify *VK055*_3379 for qRT-PCR
Pr18435	GGCGGCGTTTACTGATGTTG	Forward primer to amplify *VK055*_3380 for qRT-PCR
Pr18436	GTTAAGCATCCAGGCGTTCG	Reverse primer to amplify *VK055*_3380 for qRT-PCR
Pr18437	GATCGCTATGCGGTGGAACT	Forward primer to amplify *VK055*_0304 for qRT-PCR
Pr18438	ATCCCGCACATAGGAGGAGA	Reverse primer to amplify *VK055*_0304 for qRT-PCR
Pr18439	CCTTAGAACGAGCCACCGAA	Forward primer to amplify *VK055*_0305 for qRT-PCR
Pr18440	AGCCTCTTCATACTGTCGCC	Reverse primer to amplify *VK055*_0305 for qRT-PCR
Pr18441	TAATCTGGTGGCCGGGTTTC	Forward primer to amplify *VK055*_4482 for qRT-PCR
Pr18442	GACTAATCGCCCAGGAGAGG	Reverse primer to amplify *VK055*_4482 for qRT-PCR
Pr18443	ACGCTTTCGGCTATTTTGGC	Forward primer to amplify *VK055*_4483 for qRT-PCR
Pr18444	ACGCCATCCGCTTTGATTTG	Reverse primer to amplify *VK055*_4483 for qRT-PCR
Pr18093	GATTATTTTTTTATTATGGTCTAAAGCAG ATGAGATCCGCCAAAAACATAAAG	Forward primer to amplify the backbone of pTH15802
Pr18094	CTGTATAAGGTGCAATCAATGAGGCATTT GAGAAGCACACGG	Reverse primer to amplify the backbone of pTH15802
Pr18095	CCGTGTGCTTCTCAAATGCCTCATTGATTG CACCTTATACAG	Forward primer to amplify the promoter of *rmpADC*
Pr18096	CTTTATGTTTTTGGCGGATCTCATCTGCTT TAGACCATAATAAAAAAATAATC	Reverse primer to amplify the promoter of *rmpADC*
Pr16889	CATGCCATGGTGCAAGAGAATTATAAGATT CTGGTTGTGGATG	Forward primer to amplify *ompR* with NcoI site
Pr16890	CCCAAGCTTTGCCTTAGAACCGTCCGGGA CGAAG	Reverse primer to amplify *ompR* with HindIII site
Pr18079	CCGTGTGCTTCTCAAATGCCATGAGATCCG CCAAAAACATAAAG	Forward primer to amplify the luciferase gene
Pr18062	CCTGAGCAAACTGGCCTCATTACAATTTGG GCTTTCCGC	Reverse primer to amplify the luciferase gene
Pr18063	GCGGAAAGCCCAAATTGTAATGAGGCCAG TTTGCTCAGG	Forward primer to amplify the backbone from pTH15652
Pr18080	CTTTATGTTTTTGGCGGATCTCATGGCATTT GAGAAGCACACGG	Reverse primer to amplify the backbone from pTH15652
Pr18325	CAATGATGAGCAGCATAGTGATG	The forward primer of P*atp*1 (421 bp) for EMSA
Pr18326	CCTGCTCCCTTTGAGGTATG	The reverse primer of P*atp*1 and P*atp*2 for EMSA
Pr19387	ACAGAACGCTGAAATGCACG	The forward primer of P*atp*2 (183 bp) for EMSA
Pr19385	CCGCGTCACACTTCTTTCAC	The forward primer of P*gcv* (212 bp) for EMSA
Pr19386	CATTAACGGCGCGACAATTAG	The reverse primer of P*gcv* for EMSA

"_": BsaI site.
